# The Australian Traumatic Brain Injury Initiative: Review and Recommendations for Outcome Measures for Use With Adults and Children After Moderate-to-Severe Traumatic Brain Injury

**DOI:** 10.1089/neur.2023.0127

**Published:** 2024-04-11

**Authors:** Jennie L. Ponsford, Amelia J. Hicks, Matthew K. Bagg, Ruby Phyland, Sarah Carrier, Amelia C. James, Natasha A. Lannin, Nick Rushworth, Terence J. O'Brien, Peter A. Cameron, D. Jamie Cooper, Regina Hill, Belinda J Gabbe, Melinda Fitzgerald

**Affiliations:** ^1^ Monash-Epworth Rehabilitation Research Centre, Epworth Healthcare, Melbourne, Victoria, Australia.; ^2^ School of Psychological Sciences, Monash University, Melbourne, Victoria, Australia.; ^3^ Curtin Health Innovation Research Institute, Faculty of Health Sciences, Curtin University, Bentley, Western Australia, Australia.; ^4^ Perron Institute for Neurological and Translational Science, Nedlands, Western Australia, Australia.; ^5^ Centre for Pain IMPACT, Neuroscience Research Australia, Sydney New South Wales, Australia.; ^6^ Department of Neuroscience, Central Clinical School, Monash University, Melbourne, Victoria, Australia.; ^7^ Alfred Health, Melbourne, Victoria, Australia.; ^8^ Brain Injury Australia, Sydney, New South Wales, Australia.; ^9^ School of Public Health and Preventive Medicine, Monash University, Melbourne, Victoria, Australia.; ^10^ Emergency and Trauma Centre, The Alfred Hospital, Melbourne VIC 3004, Australia.; ^11^ Department of Intensive Care and Hyperbaric Medicine, The Alfred Hospital, Melbourne Victoria, Australia.; ^12^ Regina Hill Effective Philanthropy Pty Ltd., Melbourne, Victoria, Australia.; ^13^ Health Data Research UK, Swansea University Medical School, Swansea University, Singleton Park, United Kingdom.

**Keywords:** common data elements, outcome measures, review, traumatic brain injury

## Abstract

The Australian Traumatic Brain Injury Initiative (AUS-TBI) aims to select a set of measures to comprehensively predict and assess outcomes following moderate-to-severe traumatic brain injury (TBI) across Australia. The aim of this article was to report on the implementation and findings of an evidence-based consensus approach to develop AUS-TBI recommendations for outcome measures following adult and pediatric moderate-to-severe TBI. Following consultation with a panel of expert clinicians, Aboriginal and Torres Strait Islander representatives and a Living Experience group, and preliminary literature searches with a broader focus, a decision was made to focus on measures of mortality, everyday functional outcomes, and quality of life. Standardized searches of bibliographic databases were conducted through March 2022. Characteristics of 75 outcome measures were extracted from 1485 primary studies. Consensus meetings among the AUS-TBI Steering Committee, an expert panel of clinicians and researchers and a group of individuals with lived experience of TBI resulted in the production of a final list of 11 core outcome measures: the Functional Independence Measure (FIM); Glasgow Outcome Scale-Extended (GOS-E); Satisfaction With Life Scale (SWLS) (adult); mortality; EuroQol-5 Dimensions (EQ5D); Mayo-Portland Adaptability Inventory (MPAI); Return to Work /Study (adult and pediatric); Functional Independence Measure for Children (WEEFIM); Glasgow Outcome Scale Modified for Children (GOS-E PEDS); Paediatric Quality of Life Scale (PEDS-QL); and Strengths and Difficulties Questionnaire (pediatric). These 11 outcome measures will be included as common data elements in the AUS-TBI data dictionary.

Review Registration PROSPERO (CRD42022290954).

**Figure:**
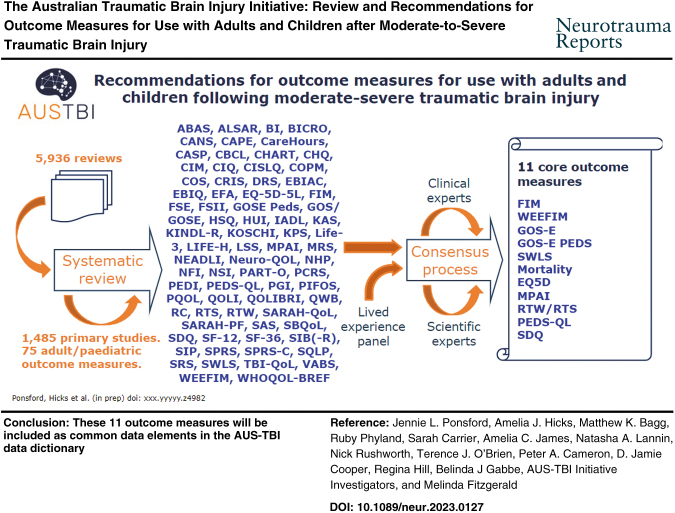


## Introduction

Moderate-to-severe traumatic brain injury (TBI) is a leading cause of disability and mortality worldwide.^1^ TBI is a complex and heterogeneous condition, associated with debilitating sequelae across numerous areas of functioning.^2–4^ The effects of moderate-to-severe TBI commonly persist for many years.^2,4,5^ Although the significant and long-term impact of moderate-to-severe TBI on outcomes is now well established, knowledge advancement has been hindered by a lack of consistency in outcome measurement. Although Australian researchers have contributed significantly to the international body of TBI management and outcomes literature, there is no uniform nationwide collection of TBI data.

The majority of studies documenting outcomes following moderate-to-severe TBI in Australia have involved individuals presenting to specific trauma centers or those receiving rehabilitation, most commonly in urban regions in one or two states.^2,6–8^ It is not known how applicable these outcome measures are to individuals treated in rural areas, those not receiving rehabilitation, and individuals from different cultural backgrounds including Aboriginal and Torres Strait Islander groups, who may be particularly vulnerable to TBI.^9^

To advance knowledge of TBI outcomes within Australia and enable comparison of outcomes with those of individuals with TBI in the rest of the world, it is critically important that studies use consistent outcome measures. This consistency may be best achieved through development of common data elements. Common data elements for TBI research have been identified by consensus in other regions, including the United States and Europe,^10,11^ but may not necessarily meet the needs of the Australian context.^12^

There have been two previous Australian initiatives to produce common data elements. Through a comprehensive literature search, Tate and coworkers^13^ identified 728 unique measures of cognitive, behavioral, social, and functional outcomes, highlighting 70 more commonly used ones and classifying them according to the International Classification of Functional Disability and Health (ICF) dimensions. Honan and coworkers^14^ identified through nomination, literature search, and international expert opinion, 115 measures in categories of: global outcome, communication, social cognition, behavioural and executive function, other neuropsychological functioning, psychological status, TBI-related symptoms, activities and participation, support and relationships, sense of self, and health-related quality of life (QoL), with final recommendations of 56 instruments for use in early recovery, outcome, and intervention studies. A parallel set of recommendations for pediatric measures was led by Wearne and coworkers.^15^ These studies, which pre-dated this project by 5–10 years, provide useful background on measures applicable to TBI, but the selection of measures was not based on a systematic literature search. Moreover, a smaller set of measures would be needed for a nationwide TBI outcomes study.

The Australian Traumatic Brain Injury Initiative (AUS-TBI) is an Australian government–funded initiative that aims to develop a set of common data elements suitable for the Australian context. This specific initiative aimed, through collaborative consultation involving expert researchers, clinicians, Aboriginal and Torres Strait Islander representatives, and individuals with TBI, to select a set of measures to predict and assess outcomes for adults and children with TBI across Australia, with the ultimate aim of improving management and rehabilitation for adults and children who have sustained a moderate-to-severe TBI.^12^ To meet these aims, the common data elements needed to cover the trajectory of a person's journey, from injury to reintegration back into the community; be relevant to persons from all states and territories in Australia in both metropolitan and rural areas and to broad demographic groups; and span the diversity of clinical presentations of persons with moderate-to-severe TBI, including persons with other injuries and/or comorbidities.^13^ It was also seen as important that selected measures show evidence of validity and reliability, sensitivity to change over time and in response to interventions; have potential to be collected in a timely fashion at low cost, via telephone, from individuals with TBI, close others, and/or clinicians; and have been translated into different languages and used internationally. The objective of this review was to use an evidence-based consensus approach to develop up-to-date recommendations for AUS-TBI for outcome measures to be used for pediatric and adult moderate-to-severe TBI based on these criteria.

## Methods

### AUS-TBI Steering Committee and Expert Panel

The identification of outcome measures was conducted as a collaboration among the AUS-TBI Steering Committee, an expanded expert panel of 30 TBI clinicians and researchers across a range of disciplines and cultural groups, and people with living experience of TBI. The group included individuals with expertise in TBI care, including those working in acute care, rehabilitation, and community settings, in both child and adult services.^14^ The lived experience group was represented on the expert panel by the CEO of Brain Injury Australia, Nick Rushworth, who is also a person with lived experience of TBI. Although there were also representatives from the Aboriginal and Torres Strait Islander community, given the important cultural consideration, AUS-TBI established an Aboriginal and Torres Strait Islander Advisory Group that met several times separately and considered the data elements and provided recommendations regarding the ways of collecting data and considerations for the project approach specific to Aboriginal and Torres Strait Islander Peoples. These processes and recommendations are outlined in another article.^16^

### Comprehensive review

#### Selection of outcome domains

A broad list of outcome domains was initially selected in consultation with the AUS-TBI Steering Committee and the expert panel, guided by the World Health Organization (WHO ICF) core set of outcome data elements.^17^ Preliminary pilot database searches encompassing the WHO ICF Core set of TBI outcome domains produced an extremely large number of records (i.e., 250,000–1,000,000 depending on search parameters). To develop an achievable review, a consensus decision was reached to (1) focus on outcomes of mortality, morbidity, everyday function, and QoL, and (2) conduct a systematic review of reviews. This comprehensive review was conducted in three key stages; (1) systematic review of reviews; (2) identification of primary studies within each review; (3) extraction of outcome measures from within the primary study.

## Stage 1. Systematic Review of Reviews

This review was prospectively registered on the International Prospective Register of Systematic Reviews (PROSPERO, CRD42022290954). Our methodology was guided by best practice systematic review methodology (i.e., as per Preferred Reporting Items for Systematic Reviews and Meta-Analyses [PRISMA]^18^); however, it did not strictly adhere to this because of the very broad scope and objectives of our review as outlined in the next sections.

### Search strategy and information sources

Standardized and piloted searches were conducted in Cumulated Index to Nursing and Allied Health Literature (CINAHL), Cochrane Database of Systematic Reviews, Embase, Epistemonikos, MEDLINE^®^, PsycINFO, PubMed, and SPORTDiscus. Searches were from inception through April 3, 2022 (search strategies provided in Supplementary Text), and were limited to the English language because of the Australian context. Keywords were collected through experts' opinion, literature review, controlled vocabulary (CINAHL Headings, Medical Subject Headings [MeSH], Excerpta Medica Tree [EMTREE], APA Thesaurus, and Sports Thesaurus), and review of the primary search results. We used Canadian Agency for Drugs and Technologies in Health's (CADTH) systematic review search filters for Embase, MEDLINE, PsycINFO, and PubMed. We also applied University of Texas School of Public Health's systematic review search filter for CINAHL and its modified version for SPORTDiscuss. As the Cochrane Database of Systematic Reviews contains only systematic reviews, we did not add a systematic review block for this source. We used the default filtering options in Epistemonikos's search interface to only retrieve the secondary studies. Search results were de-duplicated in EndNote X9 and then uploaded to Covidence for screening.

### Inclusion Criteria

Reviews were selected for inclusion according to the following criteria.

#### Studies

We included both narrative and systematic reviews.

#### Participants

Eligible reviews included studies of participants of any age and sex who had sustained moderate-to-severe TBI (penetrating or non-penetrating) of any cause. Moderate-to-severe TBI was defined as the reported presence of at least one of the following: (1) initial or lowest Glasgow Coma Scale <13, (2) post-traumatic amnesia duration >24 h, or (3) abnormal findings on computed tomography (CT) imaging of the head. This operational definition included the complicated-mild injury type. There were no restrictions on age at injury or time since injury. Reviews sampling participants without TBI, or with mild uncomplicated TBI were included under two provisions. Either the data on participants with moderate-to-severe TBI were reported separately from that of other participants or, >80% of the sample were people with moderate-to-severe TBI. Reviews with self-reported, non-medical informants or historical diagnoses of TBI were excluded. Studies without clearly reported medical confirmation of moderate-to-severe TBI were included on the expert clinical judgement of the review team that medical confirmation would have necessarily occurred in the study context. There were no restrictions on the settings in which the injury or study processes occurred.

#### Interventions

Reviews reporting on any interventions were considered for inclusion.

#### Comparators

Eligible reviews compared any interventions with any types of comparators. There were no restrictions on the type of comparator; placebo, active control (e.g., drugs within the same pharmacological class or another class), supportive, standard care, or a non-pharmacological intervention were all accepted. Studies with no comparator were also eligible for inclusion.

#### Outcomes

As was described, the original scope of the review was to examine all outcome domains identified in the WHO ICF core set of outcome data elements.^17^ This included functional outcome, but also specific domains of physical function, cognition, behavior, and emotional and social function. However, piloting this search with the information specialist produced an unmanageable number of records. It was decided by consensus with the AUS-TBI Steering Committee and Expert Panel to restrict the scope of the outcome measures to:

- Mortality
- Global outcome
- Functional outcome
- Disability
- Activities of daily living (ADL)
- Care support needed
- Return to work (RTW); return to study (RTS)
- Participation
- QoL
- Satisfaction with life

#### Study selection

Records were screened against the sampling criteria in two stages: (1) all records based on title and abstract alone, and (2) full text of all potentially eligible records. Review team members were trained to apply the eligibility criteria and have clinical area expertise and prior experience conducting systematic reviews. Title and abstract screening was completed by two independent reviewers in duplicate (A.J.H., S.C., R.P.). Disagreements were resolved through consensus, and if required, a third team member adjudicated (A.J.H., S.C., R.P., J.L.P.). The full-length of potentially eligible records was retrieved using institutional subscriptions, loan requests, or open sources. Full-text screening was completed by one reviewer (A.J.H., S.C., R.P.). Record authors were not contacted to clarify eligibility criteria.

## Stage 2. Identification of Primary Studies from Each Review

Eligible primary studies were selected from reviews by one reviewer (A.J.H., S.C., R.P.). The inclusion criteria for primary studies were the same as those for the review stage outlined. With the exception of type of studies, we excluded reviews, case studies/case reports, and case series (*n* ≤ 9). The citation information was obtained from the review reference list. Each primary study was individually obtained using university library search engines or Google Scholar. Primary studies were then uploaded into Paperpile for data extraction.

## Stage 3. Extraction of Outcome Measures from Primary Studies

The data to be extracted about each measure was decided upon by the AUS-TBI Steering Committee. This information included the following:

- Characteristics of measure
  ○ Measure name and common abbreviation
  ○ Description of what the measure assesses
  ○ Administration time
  ○ Number of items
  ○ Cost of use
  ○ Respondent (e.g., patient, clinician, family/carer)
  ○ Independence (i.e., can the measure be completed by the person independently or is it administered via an interview format)
  ○ Method of completion (i.e., in person, via telephone, via post, online; multiple responses permissible)
  ○ Training required to administer
  ○ Languages/ translations
  ○ Psychometric properties
- Use of the outcome measure in primary studies included in review
  ○ Number of primary studies in which the measure was used
  ○ Types of primary studies in which the measure was used (i.e., observational, interventional)
  ○ Time post-injury the measure had been used (i.e. acute [inpatient]; < 3 months, 3–12 months, 1–5 years; > 5 years)
  ○ Patient population for which the measure has been used (i.e., adult, pediatric, adult and pediatric)
  ○ Whether the measure was sensitive to change over time
  ○ Whether the measure was sensitive to change following intervention

For each primary study, data were extracted by one reviewer (A.J.H., S.C., R.P.), Standardized data sheets were built in Google Sheets (GSuite, Monash University), piloted, and then adapted to the review requirements. Data items were operationalized as categorical variables where possible, and as semi-structured free text otherwise. Data that were missing, unclear in the report, or not applicable were all marked “NA” in the respective cell. Study authors were not contacted to request missing or to clarify uncertain data for this iteration of the review. Where further information was required to characterize a measure, we conducted searches of Web sites including (https://www.sralab.org/; https://www.tbims.org/list.html). The list of outcome measures and characteristics relating to each of the selection criteria was synthesized in evidence tables.

### Consensus meetings

The evidence tables were presented to the Clinician and Researcher Expert Panel in four meetings held between May and July 2022 over videoonference. The discussions were led by a professional facilitator.

#### Clinician and Researcher Expert Panel meetings

The goal of these consensus meetings was to reach agreement on the outcome measures that would feasibly and reliably capture as many relevant aspects of outcome as possible, from the perspectives of the individual with TBI and their close others within a limited time frame, through multiple modalities (in person, telephone), and ideally across cultural and linguistic groups.^18^ Evidence tables were circulated to all team members before meetings. Guided by an independent facilitator, in the first meeting, expert committee members each ranked outcome measures from the evidence tables regarding their suitability for inclusion in the data set as Tier 1 (definitely suitable), Tier 2 (possibly suitable), or Tier 3 (not suitable), based on characteristics of the outcome measure captured in the evidence tables (frequency of use in studies, psychometric properties, sensitivity to intervention, time, cost and method of administration, international use, translation) and expert opinion. Agreement and dissent with each nomination were recorded. The consensus expert opinion for each measure was used to iteratively produce a short list of Tier 1, Tier 2, and Tier 3 measures. Committee members discussed some additional measures of specific functions that were not within the scope of the search. In the second meeting held in late June 2022, a short list of measures (Tier 1) was presented, based on agreed rankings from the first meeting. There was further debate around ensuring that the list included measures that covered the full range of aspects of function across the spectrum of time post-injury, were consistent with perspectives identified by the Living Experience group; could be administered in a timely and cost-effective fashion via telephone, to adults and/or children; and that the final list also included measures that enabled international comparison.

#### Living Experience expert panel

**FIG. 1.**
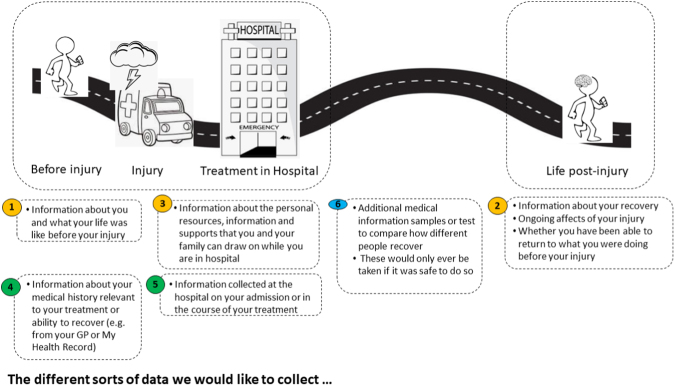
Framework for discussion with Living Experience Panel

The Living Experience Advisory Group was recruited by Connectivity Traumatic Brain Injury Australia, with assistance from the Curtin University Involve Team. The group was drawn from a database of people with living experience of TBI who had volunteered to be involved in consultations of this nature, with additional members recommended by investigators in the AUS-TBI consortium. The group included five males and four females, from four states of Australia. The researchers were advised by a representative of the body representing Australians with brain injury – someone with lived experienced of TBI – that presenting the details of many outcome measures to members of the Living Experience consumer panel could potentially be overwhelming. Rather, the aim of the consultation was to capture their views more broadly. Consultation with living experience experts was conducted across two meetings to consider the information that they thought would reflect meaningful outcomes, and the means by which they should be administered (e.g. collected about them from medical files, relatives, by phone/zoom/in person; what was an appropriate length for interviews) and how consent should be obtained. The discussions, which also covered potential predictive factors, were guided by a diagram (Fig. 1). These views were considered in the selection of measures and the final set of selected measures was circulated to the Living Experience Panel for their feedback and approval.

## Results

### Literature search

The literature search produced 5936 records. There were 4963 records excluded during the title and abstract screening stage. Of the 954 records reviewed as full text, 430 were excluded. There were 520 reviews deemed eligible for inclusion.

### Identification of primary studies from each review

We reviewed 340/520 records to identify primary studies. This stop-point was made by team consensus and reflected satisfactory data saturation, as 34 records reviewed had not contributed any new primary studies. We identified 1485 primary studies from the 340 reviews.

### Extraction of outcome measures from primary studies

We reviewed 568/1485 of the primary studies. Of the 568 primary studies reviewed, 149 were excluded as being not eligible for inclusion. Data were extracted from 419 primary studies. This stop-point was made by team consensus and reflected satisfactory data saturation, as primary studies were no longer contributing new measures. The previous 10 new measures identified were specific to the primary study and presumably had been generated by the research team, as no information could be identified for the measure. We extracted information for 75 outcome measures (see Table S1).

## Consensus Meetings

### Clinician and Researcher Expert Panel

The list of outcome measures arising from the systematic review process (Supplementary Text) was discussed by the Clinician and Researcher Expert Panel and refined to a short list of 11 via the iterative series of consensus meetings. This list is outlined subsequently and summarized in Table 1.

**Table 1.** Final Core Outcome Measures

Participant group	Measure name (abbreviation)	Administration (items; time; cost; respondent)	Psychometric properties	Languages	Sensitive to intervention^1^
	Mortality				
Adult	Functional Independence Measure (FIM)	20-30 min 18 items Not free Clinician	Adult TBI population Concurrent validity • Minutes of nursing care at admission: *r* = -0.54 (FIM Motor)^32^ • Minutes of nursing care at discharge: *r* = -0.51 (FIM Motor)^32^ • Minutes of nursing care at admission: *r* = -0.35 (FIM Cognitive)^32^ • Minutes of nursing care at discharge: *r* = -0.47 (FIM Cognitive)^32^ • Need for physical assistance: sensitivity 79%, specificity 86% (FIM Motor)^33^ Other adult clinical populations Inter-rater and intra-rater reliability • Two independent physiotherapists^34^: ○ Total score: ICC = 0.83 ○ Individuals items: *k* = 0.14 - 0.70 (*k* ≥ 0.6 for 8/13 Motor items; 0/5 Cognitive items; *k* < 0.4 for 1/13 Motor items; 4/5 Cognitive items) Concurrent validity • Expanded Disability Status Scale: *r* = -0.91 (FIM Total)^34^ • OPCS: *r* = 0.82 (FIM Total)^35^^2^ • OPCS: *r* = 0.84 (FIM Motor)^35^ • OPCS: *r* = 0.43 (FIM Cognitive)^35^ • WAIS-R-VIQ: *r* = 0.35 (FIM Total)^35^ • WAIS-R-VIQ: *r* = 0.27 (FIM Motor)^35^ • WAIS-R-VIQ: *r* = 0.51 (FIM Cognitive)^35^ Internal consistency • Cronbach coefficient alpha = 0.95 (FIM Total)^35^ • Cronbach coefficient alpha = 0.95 (FIM Motor)^35^ • Cronbach coefficient alpha = 0.89 (FIM Cognitive)^35^ Convergent and divergent validity • Higher correlation with similar constructs^35^ ○ OPCS: *r* = 0.82 (FIM Total) • Lower correlation with dissimilar constructs^35^ ○ LHS: *r* = 0.32 ○ GHQ-28: *r* = 0.13	Available in multiple languages	Yes
	Glasgow Outcome Scale/ Glasgow Outcome Scale-Extended (GOS/GOS-E)	15 min 19 items^3^ Free Clinician	Adult TBI population GOS Test-retest reliability • Two weeks (both postal surveys): *k_w_* = 0.94^36^ • Two weeks (postal and phone survey): *k_w_* = 0.67^36^ Inter-rater and intra-rater reliability • Two independent raters: >95%^37^ • Psychologist and relative: *k_w_* = 0.79^38^ • Psychologist and medical practitioner: *k_w_* = 0.45^39^ Concurrent validity • Neurobehavioural Rating Scale – Revised: *r* = 0.72^40^ GOS-E Test-retest reliability • Two weeks (two postal surveys): *k_w_* = 0.98^36^ • Two weeks (postal survey and phone interview): *k_w_* = 0.92^36^ • ≤ 1 month (phone interview and in-person interview): *k_w_* = 0.84 – 0.92^39^ Inter-rater and intra-rater reliability • Two independent raters: *k_w_* = 0.85^40^ Concurrent validity • Disability Rating Scale: *r* = -0.89; Barthel Index: *r* = 0.46; duration of post-traumatic amnesia: *r* = 0.52.^41^	Available in multiple languages	Yes
	Satisfaction with Life Scale (SWLS)	< 5 min 5 items Free Patient	Adult TBI population Concurrent validity • Community Integration Questionnaire-Revised: *r* = 0.34^42^ Other adult non-clinical populations Test-retest reliability • Two months: *r* = 0.82^26^ Internal consistency • Cronbach coefficient alpha = 0.87^26^ Convergent and divergent validity • Higher correlation with similar constructs^26^ ○ Andrews/Withey Scale: *r* = 0.68^26^ ○ Fordyce Global Scale: *r* = 0.58^26^ ○ Fordyce Global Scale: *r* = 0.68^43^ ○ LSI-A: *r* = 0.82^43^ • Lower correlation with dissimilar constructs^44^ ○ BDI: *r* = -0.72	Available in multiple languages	Yes
Adult & Pediatric	EuroQol-5 Dimensions, five-level version (EQ-5D-5L)	< 10 min 5 items Not free Patient or family/carer or clinician	Other adult clinical populations Test-retest reliability • 2 weeks: ICC = -0.76 (total score)^45^ Convergent validity • Dementia specific QoL measures: *r* = 0.11 - 0.49 (total score) ^46^ Other adult mixed clinical and non-clinical populations Test-retest reliability • 1-3 weeks: *k* = 0.68^45,47^ • 1-3 weeks: *k_w_* = 0.54^45^ Convergent validity • Multi-attribute utility instruments: pooled rho = 0.76^28^ • Physical/functional measures: pooled rho = 0.5^28^ • Pain/discomfort measures: pooled rho = 0.60^28^ • Satisfaction measures: pooled rho = 0.34^28^ • Cognition/communication: pooled rho = 0.26^28^	Available in multiple languages	Yes
	Mayo Portland Adaptability Inventory-4 (MPAI-4)	8-10 min 29 items^5^ Free Patient or family/carer or clinician	Adult TBI population Concurrent validity • Disability Rating Scale: *r_s_* = 0.81 (MPAI-Version 1)^48^ Predictive validity • Assessment at discharge within 1 year to independent living: *r* = -0.64 (MPAI-22)^48^ • Assessment at discharge within 1 year to vocational outcome: *r* = -0.37 (MPAI-22)^49^ Convergent and divergent validity • Higher correlation with similar constructs^49^ ○ RAVLT: *r* = -0.55 (Cognitive Index; MPAI-Version 1) ○ WCST: *r* = 0.56 (Cognitive Index; MPAI-Version 1) • Lower correlation with dissimilar constructs^49^ ○ RAVLT: *r* = -0.22 (Non-Cognitive Index; MPAI-Version 1) ○ WCST: *r* = 0.29 (Non-Cognitive Index; MPAI-Version 1)	Available in multiple languages	Yes
	Return to study/ return to work (RTS/ RTW)	Variable Variable Free Patient or family/carer or clinician	N/A	Any	Yes
Pediatric	Functional Independence Measure for Children (WEEFIM)	15-20 min 18 items Not Free Clinician	Other pediatric clinical populations Test-retest reliability • 14 days: ICC = 0.97 (Total WEEFIM)^50^ • 14 days: ICC = 0.98 (Motor WEEFIM)^50^ • 14 days: ICC = .97 (Cognitive WEEFIM)^50^ • ≤ 21 days (interview and observation): ICC = 0.93 (Total WEEFiM) Discriminant validity • Significant difference in total score between a group with disabilities and a group without disabilities^50^	Available in multiple languages	Unknown
	Glasgow Outcome Scale- Extended Paediatric Revision (GOS Peds)	15 min 1 Item^8^ Free Clinician	Pediatric TBI patients Concurrent validity • VABS: *r* = -0.65 to -0.75^31^ Predictive validity • VABS: *r* = -0.69 (3 months)^31^ Discriminant validity • CPRS: *r* = -0.009 to 0.46^31^	Available in multiple languages	Unknown
	Paediatric Quality of Life Inventory (PedsQL)	4 min 23 items Not Free Patient or family/ carer	Pediatric TBI patients Test-retest reliability • 9 days (average): *r* = 0.75-0.90 (range across subscales and total score)^51^ Concurrent validity • BRIEF: *r =* -0.10-0.70 (range across subscales and total score)^51^ Internal consistency • Cronbach coefficient alpha = 0.74-0.93 (range across subscales and total score)^51^ Discriminant validity • PedsQL Cognitive Functioning Scale strongly discriminated children by head AIS severity and parents' assessment of change in cognition score^52^ Other pediatric mixed clinical and non-clinical populations Internal consistency • Cronbach coefficient alpha = 0.53-0.85 (range across subscales and total score)^52^	Available in multiple languages	Unknown
	Strengths and Difficulties Questionnaire (SDQ)	5-10 min 25 items Free Patient or family/carer or clinician or teacher	Other pediatric non-clinical populations Test-retest reliability • Two months: *r* = 0.75-91 (parent report; range across subscales)^53^ • Two months: *r* = 0.71 (self-report; total score)^54^ • Two months: *r* = 0.59-87 (self-report; range across subscales)^54^ • Four to six months: r = 0.62 (self-report, total score)^55^ • Four to six months: r = 0.72 (parent report, total score)^54^ • Four to six months: r = 0.80 (teacher report, total score)^54^ Inter-rater and intra-rater reliability • Parent and teacher: *r* = 0.41 (total score)^56^ • Parent and teacher: r = 0.46 (total score^55^ • Parent and youth: r = 0.48 (total score)^56^ • Youth and teacher: r = 0.44 (total score)^55^ Concurrent validity • CBCL: *r* = 0.70 (parent report, total score) (Muris, Meesters, and van den Berg 2003)^54^ • CBCL: *r* = 0.72 (parent report, total score) (Mieloo et al. 2012)^56^ • CBCL: *r* = 0.76 (teacher report, total score) (Mieloo et al. 2012)^56^ • CDI-P: *r* = 0.73 (parent report, total score) (Muris, Meesters, and van den Berg 2003)^54^ • ADHDQ-P: *r* = 0.67 (parent report, total score) (Muris, Meesters, and van den Berg 2003)^54^ Internal consistency • Cronbach coefficient alpha = 0.81 (self-report; total score) (Yao et al. 2009)^53^ • Cronbach coefficient alpha = 0.80 (self-report; total score) (Goodman 2001)^55^ • Cronbach coefficient alpha = 0.64 (self-report; mean across subscales) (Muris, Meesters, and van den Berg 2003)^54^ • Cronbach coefficient alpha = 0.70 (parent report; mean across subscales) (Muris, Meesters, and van den Berg 2003)^54^ • Cronbach coefficient alpha = 0.77 (parent report; total score) (Mieloo et al. 2012)^56^ • Cronbach coefficient alpha = 0.82 (parent report; total score) (Goodman 2001)^55^ • Cronbach coefficient alpha = 0.81 (teacher report; total score) (Mieloo et al. 2012)^56^ • Cronbach coefficient alpha = 0.87 (teacher report; total score) (Goodman 2001)^55^	Available in multiple languages	Unknown

ADHDQ-P, Attention-Deficit/Hyperactivity Disorder Questionnaire – Parent; BDI, Beck Depression Inventory; BRIEF, Behavior Rating Inventory of Executive Function; CBCL, Child Behavior Checklist; CDI-P CPRS ICC, intraclass coefficient; LSI, Live Satisfaction Index; OPCS RAVLT, Rey Auditory Verbal Learning Test; TBI, traumatic brain injury; VABS, Vineland Adaptive Behavior Scales; WCST, Wisconsin Card Sorting Test.

^1^ A measure was categorized as “sensitive to intervention” if there was a significant change recorded on the measure from pre- to post-intervention.

^2^ Hobart et al 2001 did include nine participants with TBI (4% of the sample). As such, psychometric properties from this study are reported under Other Adult Clinical Populations

^3^ GOSE has a 19-item interview schedule to assist the clinician in categorizing the patient's outcome; however, outcome is ultimately rated on a single item.

^4^ 56% of sample in Reisetter et al (2005) had sustained a TBI. As such, psychometric properties from this study are reported under Adult TBI Patients.

^5^ The MPAI-4 comprises 29 core items that are scored, and 6 additional items that are not scored.

^6^ 80% of sample in Malec et al (1994) had sustained a TBI. As such, psychometric properties from this study are reported under Adult TBI Patients

^7^ 72% of sample in Malec et al (2001) had sustained a TBI. As such, psychometric properties from this study are reported under Adult TBI Patients

^8^ GOS-E Peds has a seven-item interview schedule to assist the clinician in categorizing the patient's outcome; however, outcome is ultimately rated on a single item.

#### Living Experience Expert Group

The Living Experience Expert Group met separately from the Clinician and Researcher Expert Panel, although one representative attended both meetings. The Living Experience Expert Group expressed the following views.

- Data collected need to be personally meaningful and helpful for the person themselves, with an inclusive focus. It was understood that the person with TBI may not always be able to give consent. However, it was considered important to ensure that the person providing consent be the most appropriate person to do so, from the perspective of the injured person, and that this may change over time.
- It was agreed that both pre-injury and post-injury information was needed to capture the changes and loss of continuity of life post-injury. The panel members felt that cognitive and communication changes, as well as emotional, social, and family factors were important, in additional to medical information. Fatigue and sexual function were also seen as important issues to capture. The panel considered that the most important outcome information should show the connection between function and life after the injury, compared with pre-injury function, including activities, work, and other aspects of life, and how the person feels about this.
- When collecting information regarding recovery and outcome it was seen as important to do the following.
  ○ Make it very clear why the questions are being asked, how the information will be used, and that responses will not affect care given to the person with TBI.
  ○ Provide different ways to connect with people, with options of online data collection, telephone, and video calls being important. Build rapport so the person giving the information feels comfortable and handle difficult issues sensitively. The way questions are asked is very important and requires good interpersonal skills and understanding of TBI as well as sensitivity to the cognitive function and circumstances of the individual with brain injury. Timing the calls with carer visits could assist.
  ○ Be aware that the questions may evoke emotions, and that referral to support systems after questioning may be required, particularly for the psychological questions.
  ○ Be aware that families may perceive recovery differently from the person with TBI. Both perspectives are important, particularly if there is a lack of awareness in the person with TBI. Different family members may view recovery differently.

These views were taken into consideration in the selection of the measures. A summary and description of the final set of measures selected was sent to the Living Experience Panel for review.

### Core measures selected

#### Adult and pediatric mortality

Given that a proportion of adults and children who sustain moderate-to severe TBI die in the acute or chronic post-injury stages,^19^ mortality is an outcome that needs to be measured at both acute and longer-term time points.

#### Adult

##### Functional Independence Measure (FIM)

The FIM is a clinician-rated measure of functional disability in adults.^20^ It is a generic measure suitable for use in clinical groups accessing medical rehabilitation, both neurological and non-neurological.^21^

The FIM comprises two scales. The Motor Scale contains four subscales: self-care (eating, grooming, bathing, dressing upper and lower body, toileting), sphincter control (bladder and bowel management), transfers (to/from bed, toilet, and bath), and locomotion (walking/wheelchair and stairs). The Cognitive Scale containing two subscales: communication (language comprehension and expression) and social cognition (social interaction, problem solving and memory). Ratings are made on a seven-point scale; 1 (equivalent of total assistance – patient expends <25% of effort or is unable to perform the task); 2 (maximal assistance – patient expends 25–49% of effort), 3 (moderate assistance – patient expends 50–74% of effort), 4 (minimal assistance – patient expends ≥75% of effort), 5 (supervision or set-up), 6 (modified independence – use of a device/aid, safety or time issues), or 7 (complete independence, without modification, aid, or device, and performed in a safe and timely manner).^21^ The seven-point scale is organized into two broad levels of functioning: independence (score 6 or 7) and dependence (score 1, 2, 3, 4, or 5). The FIM total score ranges from 18 to 126 (Motor scale: 13–91, Cognitive scale: 5–35), with higher scores indicating greater independence. This measure was included because it has good psychometric properties, has shown sensitivity to change in inpatient settings (although not well over the longer term), is widely used internationally, and is available in multiple languages. Although it requires clinician training, it is routinely collected on admission and discharge by all rehabilitation hospitals Australia-wide.

##### Glasgow Outcome Scale-Extended (GOS-E)

The GOS-E is a clinician-rated measure of global outcome that is widely used with TBI groups.^21^ It represents an expansion of the original five-category GOS^22,23^ to an eight-category classification scale (dead, vegetative state, lower severe disability, upper severe disability, lower moderate disability, upper moderate disability, lower good recovery, upper good recovery).^24^ A structured interview has been developed to provide greater objectivity and reliability.^25^ The interview samples seven main areas (consciousness, independence in the home, independence outside the home [for shopping and travel], work, social and leisure activities, family and friends, and return to normal life). For all areas (with the exclusion of consciousness), the interview clarifies whether these problems were present prior to injury. For items regarding family and friends, and return to normal life, if problems were present pre-injury the clinician is asked to assess whether the problems have become “markedly worse.” Two items inquire about epileptic seizures and seizure risk. A final item clarifies what the most important factor is in the outcomes recorded: (1) effects of brain injury, (2) effects of illness or injury to another part of the body, (3) a mixture of these. This is the most widely used measure in global outcome studies internationally. It has satisfactory psychometric properties and has shown sensitivity to change in cohort and interventional studies. It can be administered within 5–10 min via telephone interview with the patient or close other and agreement with questionnaire format is acceptable.^26^

##### Satisfaction with Life Scale (SWLS)

The SWLS is a measure of global life satisfaction used widely with TBI groups.^21,26^ The items are intentionally global, to provide a rating by the individual with TBI of life satisfaction as a whole.^21,26^ Ratings are made on a -seven point scale from “strongly disagree” to “strongly agree.” The total score ranges from 5 to 35, with higher scores indicating greater satisfaction. This measure has been widely used internationally, is available in multiple languages, shows sensitivity to change in cohort and intervention studies, and can be administered in <5 min.

#### Adult and pediatric measures

##### EuroQol-5 Dimensions, five-level version (EQ-5D-5L)

The EuroQol-5D-5L is a measure of health status.^27^ The EQ-5D-5L includes five items where respondents self-report any problems in relation to mobility, self-care, daily activities, pain/discomfort, and anxiety/depression.^27^ Each dimension is described on five possible levels: (1) no problems, (2) slight problems, (3) moderate problems, (4) severe problems, and (5) extreme problems/unable to.^27^ Responses can be converted into a single measure of health utility (EQ-Index score) using preference-based (typically country-specific) weights.^28^ Health utility values generally range from 0 (death) to 1 (perfect health). This self-report measure of quality of life in adult and pediatric individuals is widely used internationally and translated into multiple languages, sensitive to change in cohort and intervention studies, can be used to examine cost-benefit of interventions, and can be administered in <5 min in person or via telephone to the person with TBI or proxy.

##### Mayo Portland Adaptability Inventory-4 (MPAI-4)

The MPAI was designed for the post-acute assessment of adults and children >1 year of age with acquired brain injury (ABI), to increase understanding of outcomes and evaluate rehabilitation programs.^28^ The MPAI-4 comprises three subscales: Ability (sensory, motor, and cognitive abilities), Adjustment (mood, interpersonal interactions), and Participation (social contacts, initiation, money management). There is a fourth subscale that is not scored: Pre-existing and associated conditions. The MPAI-4 may be completed by clinicians, informants, or self-ratings. Ratings are made on a five-point scale, with tailored response descriptors.^21,28^ Raw scores can be transformed to T scores to allow comparison with MPAI-4 standardization samples. Higher scores indicate more severe impairment and limitations. This clinician-rated measure captures information from the patient or close other regarding a broad range of physical, sensory, cognitive, communication, behavioral, emotional, and sexual symptoms or changes and participation outcomes relevant to individuals with TBI; has excellent psychometric properties; has been widely used internationally, translated into multiple languages, shown sensitivity to change over time and in response to intervention; and can be administered in 8–10 min. It provides the most comprehensive coverage of TBI-related symptoms and consequences.

##### Return to Study/ Return to Work (RTS/ RTW)

There is no standardized method to measure RTS or RTW. Common indices used include RTS: active education (attendance at school, hours per week), education level achieved post-injury; dichotomous scale: studying/not studying; need for formal support at school; structured interview to determine difficulties in RTS. For RTW common indices include: working status (attendance at work, tasks performed); dichotomous scale: working/not working; hours per week; various categorical assessments, for example, unemployed, casually employed, employed part time, employed full time, retired; type of work; job satisfaction; income; years spent remaining in employment (“survival in employment”); stability of employment; structured Interview to determine difficulties in RTW. Return to work or study is a widely used outcome across TBI studies in adults and, for return to study, in children, internationally. Although it is assessed in multiple ways, information can be standardized for international comparison, and this measure has shown sensitivity to change over time in cohort studies.

#### Pediatric measures

##### Functional Independence Measure for Children (WEEFIM)

The WEEFIM is a clinician-rated measure of functional disability in a developmental context.^29^ The items, subscales, and scales for the WEEFIM are the same as for the FIM. The WEEFIM is intended for children between 6 months and 7 years of age; however, it can be used in older age groups.^21,29^ For each item there are corresponding age-appropriate expectations. The same seven-point rating scale used by the FIM is used for the WEEFIM, with higher scores indicating greater independence. Normative data are required to interpret WEEFIM data. A normative data set is available for total scores,^30^ enabling interpretation of scores in relation to expected developmental stages. The adult FIM is routinely collected by trained clinicians in pediatric rehabilitation centers in Australia, therefore being most useful in this recovery phase. It is used internationally and translated into multiple languages. Its sensitivity to change following intervention remains undocumented.

##### Glasgow Outcome Scale Modified for Children (GOS-E Peds)

The GOS-E Peds is a developmentally appropriate clinician-rated structured interview to classify global outcome in younger patients for children <16 years of age.^31^ The GOS-E Peds uses the same eight-category classification scale as the GOS-E, and samples the same seven areas of outcome; however, some items are modified for a developmental/pediatric context. The GOS-E Peds interview does not have specific items regarding pre-injury functioning; however, it does include an overall scoring caveat instruction to raters to consider pre-morbid status. The GOS-E Peds does not include the final three GOS-E items addressing epilepsy and factors impacting outcome. The hierarchical nature of the questions and scoring is the same as for the GOS-E. The GOS-E Peds has been less widely used than its adult version, as it was developed more recently and its sensitivity to change remains undocumented. It has been used internationally and translated into multiple languages.

##### Paediatric Quality of Life Inventory (PEDS-QL)

The PEDS-QL measures health-related quality of life for children and adolescents 2–18 years of age. The PEDS-QL comprises four subscales: Physical, Emotional, Social, and School Functioning. Ratings are made on a five-point scale that captures the extent of the child's difficulties in each item from “never” to “almost always.” Higher scores indicate greater satisfaction. This parent or child-rated measure of quality of life after TBI has shown good psychometric properties, and is used internationally and translated into multiple languages. Its sensitivity to change has not yet been documented.

##### Strengths and Difficulties Questionnaire (SDQ)

The SDQ measures general psychosocial functioning in children and adolescents 4–17 years of age. It comprises five domains: hyperactivity, peer problems, emotional symptoms, conduct problems, and pro-social behaviour. Ratings are made on a three-point scale capturing the extent to which each item is “true.” A total difficulties summary score is calculated, with higher scores reflecting greater difficulties. This parent-rated measure captures the common cognitive, behavioral, psychological, and social changes associated with TBI in children. It is brief to administer, and has been used internationally and translated into multiple languages.

## Discussion

The creation of a standardized set of outcome measures by the AUS-TBI group will provide guidance as to data to collect across metropolitan, regional, and rural Australia that meaningfully reflect outcomes and quality of life following moderate-to-severe TBI. The 11 core measures, selected on the basis of a search of systematic reviews and consensus, capture various aspects of functional outcome following moderate-to-severe TBI in adults and children. These aspects include death, independence in daily activities, symptoms, and cognitive, behavioral, and psychosocial changes experienced, and participation in vocational and avocational activities considered important by individuals with TBI. They show acceptable validity and reliability psychometrically; can capture the perspectives of individuals with TBI, their close others, and clinicians in a timely fashion via telephone or video call; have been used in international studies; and can be used in different languages. All adult measures selected also had shown sensitivity to change in cohort and /or intervention studies. The pediatric measures had been more recently developed and there were no data regarding change over time. The issue of collecting data through the transition between childhood and adulthood would also need to be addressed.

The adult measures selected were all included in the previously developed, larger set of adult TBI outcome measures described by Tate and coworkers,^17^ and the GOS-E, FIM subscales, and Satisfaction with Life Scale were among core common data elements selected by Wilde and coworkers^27^ in the United States. Honan and coworkers^29^ included all measures except the FIM in their recommended measures. The FIM was included in the current study list because it is routinely collected by Australian rehabilitation hospitals and would be readily available for data collection on patients receiving rehabilitation. Regarding pediatric measures, only the PEDS-QL and Strengths and Difficulties Questionnaire were included in the set of pediatric measures recommended by Wearne and coworkers,^29^ possibly reflecting the relative recency of development and use of pediatric outcome scales such as the GOS-Peds and the Wee-FIM.

Depending on the context and funding for data collection, there will be a need to prioritize measures to maximize data capture. The implementation of these measures, or subsets of them, will create a comprehensive Australian data set but will also enable comparison and harmonization with existing international data sets. This will enable characterization of difficulties faced by individuals with moderate-severe TBI and maximize our capacity to generate prognostic models to guide models of care. The broad adoption of such common outcome measures will aim to minimize the burden on individuals with TBI and close others, allow for pooling of data to create a means of benchmarking, allow for collection of large data sets to enable modelling to enhance understanding of heterogeneity, and answer important research questions. It will also enable comparison of outcomes following interventions, with the ultimate aim of improving outcome and quality of life after moderate-to-severe TBI.

The next steps in this project could include the piloting of data collection processes to establish the feasibility of administering these measures via telephone and seeking feedback from the individuals to whom they are administered. There is also a need to investigate which of the measures are already being collected across healthcare providers, what information can be obtained via data linkage, (e.g., for RTW, social security) and what are the optimal time points for data collection. This will be followed by the creation of data collection and management systems that will be designed to track or collect selected predictive factors and indicators as well as the abovementioned outcomes. These systems will be organized to facilitate secure data collection, linkage, storage, curation, management, and analysis.

This study had several limitations. Because of time and resource constraints and the enormous number of systematic reviews, individual TBI outcome studies and measures identified in systematic reviews conducted in the past 10 years, we were unable to review all systematic reviews or all primary studies from the systematic reviews examined. However, the review process was continued until the group agreed that a point of data saturation had been reached and that no new measures were being generated. To minimize cognitive burden on the members of the Living Experience Expert panel, they did not review all measures in detail. Rather they expressed views about the nature of what should be measured and how this should be captured, and this was considered in the processes of short listing measures. A summary of the final selected measures was approved by the panel members. Further feedback from individuals with brain injury and close others will be sought when administration of measures is being piloted.

The review was limited to a focus on functional outcomes and satisfaction with life from various perspectives, rather than the broad range of ICF Core Set outcomes originally recommended by the expert panel, because the searches yielded an unmanageable number of articles for review. The panel did recommend several additional measures that focused on specific abilities or problems considered important by panel members (e.g., cognitive function, fatigue, challenging behaviors, mobility, and anxiety and depression symptoms). Sexual function was another factor considered important by one Living Experience panel member. Notwithstanding the importance of the use of measures of specific symptoms or functions commonly associated with TBI, especially in the assessment of response to specific interventions, these measures were outside of the scope of the literature search for reasons already stated. With the exception of sexuality, these constructs are however largely captured in the self-report-questionnaires selected. The time required to administer all these additional measures unfortunately precluded their inclusion in the core set. For the reader's information, these measures have been listed in Table S2. It is important to note that this list was determined on the basis of consensus only, rather than on a systematic literature search and assessment against the key criteria for inclusion of measures. The set of core data element measures that can ultimately be collected may build on existing data collection systems in operation around Australia. Ultimately, the implementation of any of the measures will depend on funding.

Nevertheless, this study is the first to have based selection of outcome measures on a systematic literature search that has allowed for a comprehensive and evidence-informed examination of TBI outcome measures that, if implemented, could create a nationwide profile of outcomes following TBI across Australia. A parallel set of studies has identified a core set of pre-injury demographic, injury-related, and post-injury factors that will be included in a data dictionary to examine factors associated with outcome, with the ultimate aim of optimizing quality of life after TBI for all Australians.

## Transparency, Rigor, and Reproducibility Summary

The literature search for this study prospectively registered on PROSPERO (CRD42022290954). The methodology was guided by best practice systematic review methodology (i.e., as per PRISMA18); however, it did not strictly adhere to this because of the very broad scope and objectives of our review as outlined. Standardized and piloted searches were conducted in CINAHL, the Cochrane Database of Systematic Reviews, Embase, Epistemonikos, MEDLINE, PsycINFO, PubMed, and SPORTDiscus. Searches were from inception through April 3, 2022, and were limited to English because of the Australian context. Keywords were collected through experts' opinion, literature review, controlled vocabulary as outlined, and review of the primary search results. Search results were de-duplicated in EndNote X9 and then uploaded to Covidence for screening. Reviews were selected for inclusion according to the criteria outlined in this article. We included both narrative and systematic reviews. Eligible reviews included studies of participants of any age and sex who had sustained a moderate-to-severe TBI or a complicated mild TBI as defined in this article. Reviews reporting on any interventions with any type of comparators were considered for inclusion.

The original scope of the review was to examine all outcome domains identified in the WHO ICF Core Set of outcome data elements.^17^ This included not only functional outcome, but also specific domains of physical function, cognition, behavior, and emotional and social function. However, piloting this search with the information specialist produced an unmanageable number of records. It was decided by consensus with the AUS-TBI Steering Committee and Expert Panel to restrict the scope of the outcome measures to mortality, functional outcome, and quality of life, as listed on page 391. Title and abstract screening was completed by two independent reviewers in duplicate. Disagreements were resolved through consensus, and if required, a third team member adjudicated. Results of data extraction are reported in this article. Potentially eligible records were retrieved in full. Full-text screening was completed by one reviewer. Study authors were not contacted to clarify eligibility criteria. Eligible primary studies were selected from reviews by one reviewer, following the same inclusion criteria as for the review stage. The data to be extracted about each measure were decided upon by the AUS-TBI Steering Committee and Expert Panel, as outlined. Where further information was required to characterize a measure, we conducted searches of Web sites including (https://www.sralab.org/; https://www.tbims.org/list.html). The list of outcome measures and characteristics was synthesized in evidence tables.

### Consensus meetings

The evidence tables were presented to the AUS-TBI Expert Panel in four meetings held between May and July 2022 over Zoom, led by a professional facilitator, using a prioritization process considering the evidence tables to determine the final set of measures. A separate meeting was held with the Living Experience Panel as described. This panel reviewed a summary of the final set of recommended measures.

## Supplementary Material

Supplemental data

Supplemental data

Supplemental data

## The Australian Traumatic Brain Injury Initiative Investigators

*Tara Alexander*, Australasian Rehabilitation Outcomes Centre & Australian Health Services Research Institute, Faculty of Business and Law, University of Wollongong, Wollongong, New South Wales, Australia; *Vicki Anderson*, Psychology Service, The Royal Children's Hospital, Melbourne, Victoria, Australia; Clinical Sciences Research, Murdoch Children's Research Institute, Melbourne, Victoria, Australia; *Ana Antonic-Baker*, Department of Neuroscience, Central Clinical School, Monash University, Melbourne, Victoria, Australia; *Elizabeth Armstrong*, School of Medical and Health Sciences, Edith Cowan University, Perth, Western Australia, Australia; *Franz E. Babl*, Department of Emergency Medicine, The Royal Children's Hospital, Melbourne, Victoria, Australia; Departments of Paediatrics and Critical Care, University of Melbourne, Melbourne, Victoria, Australia; Murdoch Children's Research Institute, Melbourne, Victoria, Australia; *Matthew K. Bagg,* Curtin Health Innovation Research Institute, Faculty of Health Sciences, Curtin University, Perth, Western Australia, Australia; School of Health Sciences, University of Notre Dame Australia, Perth, Western Australia, Australia; Perron Institute for Neurological and Translational Science, Perth, Western Australia, Australia; Centre for Pain IMPACT, Neuroscience Research Australia, Sydney, New South Wales, Australia; *Zsolt J. Balogh*, Department of Traumatology, John Hunter Hospital and University of Newcastle, Newcastle, New South Wales Australia; *Karen M. Barlow*, Acquired Brain Injury in Children Research Program, Queensland Children's Hospital, Brisbane, Queensland, Australia; Centre for Children's Health Research, University of Queensland, Brisbane, Queensland, Australia; *Judith Bellapart*, Department of Intensive Care Services, Royal Brisbane and Women's Hospital, Brisbane, Queensland, Australia; Faculty of Medicine, University of Queensland, Brisbane, Queensland, Australia; *Niranjan Bidargaddi*, Flinders Digital Health Centre, College of Medicine & Public Health, Flinders University, Adelaide, South Australia, Australia; *Erika Bosio*, Centre for Clinical Research in Emergency Medicine, Harry Perkins Institute of Medical Research, Perth, Western Australia, Australia; School of Biomedical Science & School of Medicine, University of Western Australia, Perth, Western Australia, Australia; *Peter Bragge*, BehaviourWorks Australia, Monash Sustainable Development Institute, Monash University, Melbourne, Victoria, Australia; *Michael Bynevelt*, School of Surgery, The University of Western Australia, Perth, Western Australia, Australia; Neurological Intervention and Imaging Service of Western Australia, Sir Charles Gairdner Hospital, Perth, Western Australia, Australia; *Karen Caeyenberghs*, Cognitive Neuroscience Unit, School of Psychology, Deakin University, Geelong, Victoria, Australia; *Peter A Cameron*, National Trauma Research Institute, Melbourne, Victoria, Australia; School of Public Health and Preventive Medicine, Monash University, Melbourne, Victoria, Australia; Emergency and Trauma Centre, The Alfred Hospital, Melbourne, Victoria, Australia; *Jacquelin Capell*, Australasian Rehabilitation Outcomes Centre & Australian Health Services Research Institute, Faculty of Business and Law, University of Wollongong, Wollongong, New South Wales, Australia; *Kevin E. K. Chai,* School of Population Health, Faculty of Health Sciences, Curtin University, Perth, Western Australia, Australia; Curtin Institute for Computation, Curtin University, Perth, Western Australia, Australia; *Lyndsey E. Collins-Praino*, School of Biomedicine, University of Adelaide, Adelaide, South Australia, Australia; *D. J. Jamie Cooper*, Australian and New Zealand Intensive Care Research Centre, School of Public Health and Preventive Medicine, Monash University, Melbourne, Victoria, Australia; Department of Intensive Care and Hyperbaric Medicine, The Alfred, Melbourne, Victoria, Australia; *Gill Cowen*, School of Medicine, Faculty of Health Sciences, Curtin University, Perth, Western Australia, Australia; *Louise M. Crowe,* Clinical Sciences Research, Murdoch Children's Research Institute, Melbourne, Victoria, Australia; Department of Paediatrics, University of Melbourne, Melbourne, Victoria, Australia; *Tim Cudmore*, AUS-TBI Lived Experience Advisory Group; *Jennifer Cullen*, Synapse, Brisbane, Queensland, Australia; James Cook University, Townsville, Queensland, Australia; Menzies Health Institute Queensland, Griffith University, Brisbane, Queensland, Australia; *Kate Curtis*, Susan Wakil School of Nursing and Midwifery, Faculty of Medicine and Health, The University of Sydney, New South Wales, Australia; Illawarra Shoalhaven Local Health District, Wollongong, New South Wales, Australia; Illawarra Health and Medical Research Institute, Wollongong, New South Wales, Australia; George Institute for Global Health, Sydney, New South Wales, Australia; *Anthony Delaney*, Division of Critical Care, The George Institute for Global Health, Sydney, New South Wales, Australia; Malcolm Fisher Department of Intensive Care Medicine, Royal North Shore Hospital, Sydney, New South Wales, Australia; Northern Clinical School, Sydney Medical School, University of Sydney, Sydney, New South Wales, Australia; Australian and New Zealand Intensive Care Research Centre, School of Public Health and Preventive Medicine, Monash University, Melbourne, Victoria Australia; *Graeme Dibdin*, AUS-TBI Lived Experience Advisory Group; *Sandra Eades*, Centre for Epidemiology and Biostatistics, Melbourne School of Population and Global Health, University of Melbourne, Melbourne, Victoria, Australia; School of Medicine, Faculty of Health Sciences, Curtin University, Perth, Western Australia, Australia; *Gary F. Egan,* Monash Biomedical Imaging & School of Psychological Sciences, Monash University, Melbourne, Victoria, Australia; *Daniel Y. Ellis*, Department of Trauma, Royal Adelaide Hospital, Adelaide, South Australia, Australia; Statewide South Australian Trauma Service, South Australia, Australia; School of Public Health and Tropical Medicine, James Cook University, Queensland, Australia; *Ari Ercole*, Division of Anaesthesia, University of Cambridge, Addenbrooke's Hospital, Cambridge, United Kingdom; Cambridge Centre for AI in Medicine, University of Cambridge, United Kingdom; *Daniel M. Fatovich*, Emergency Medicine, Royal Perth Hospital, University of Western Australia, Perth, Western Australia, Australia; Centre for Clinical Research in Emergency Medicine, Harry Perkins Institute of Medical Research, Perth, Western Australia, Australia; *Murray J. Fisher*, Susan Wakil School of Nursing and Midwifery, Faculty of Medicine and Health, University of Sydney, New South Wales, Australia; Royal Rehab, Ryde, Sydney, New South Wales, Australia; *Mark Fitzgerald,* National Trauma Research Institute, Melbourne, Victoria, Australia; *Melinda Fitzgerald* (Curtin Health Innovation Research Institute, Faculty of Health Sciences, Curtin University, Perth, Western Australia, Australia; Perron Institute for Neurological and Translational Science, Perth, Western Australia, Australia; *Jennifer Fleming,* School of Health and Rehabilitation Sciences, The University of Queensland, Brisbane, Queensland, Australia); *Roslyn Francis*, Department of Health, Government of Western Australia, Perth, Western Australia, Australia; *Belinda J. Gabbe*, School of Public Health and Preventive Medicine, Monash University, Melbourne, Victoria, Australia; Health Data Research UK, Swansea University Medical School, Swansea University, Singleton Park, United Kingdom; *Adelle Gadowski*, School of Public Health and Preventive Medicine, Monash University, Melbourne, Victoria, Australia; *John Gilroy*, Aboriginal and Torres Strait Islander Research, Faculty of Medicine and Health, University of Sydney, Sydney, New South Wales, Australia; *Mitchell A. Hansen,* Department of Neurosurgery, John Hunter Hospitals and University of Newcastle, Newcastle, New South Wales, Australia; *James E. Harrison*, College of Medicine and Public Health, Flinders University, Adelaide, South Australia, Australia; *Luke J. Haseler*, Curtin Health Innovation Research Institute, Faculty of Health Sciences, Curtin University, Perth, Western Australia, Australia; *Leanne Hassett*, Institute for Musculoskeletal Health & Sydney School of Health Sciences, Faculty of Medicine and Health, University of Sydney, Sydney, New South Wales, Australia; Sydney Local Health District, Sydney, New South Wales, Australia; *Sarah C. Hellewell,* Curtin Health Innovation Research Institute & School of Medicine, Faculty of Health Sciences, Curtin University, Perth, Western Australia, Australia; Perron Institute for Neurological and Translational Science, Nedlands, Western Australia, Australia; *Amelia J. Hicks*, School of Psychological Sciences, Monash University, Melbourne, Victoria, Australia; Monash Epworth Rehabilitation Research Centre, Epworth Healthcare, Melbourne, Victoria, Australia; Brain Injury Research Center, Icahn School of Medicine at Mount Sinai, New York City, New York, United States of America; *Andrew F. Hill*, College of Science, Health and Engineering, La Trobe University, Melbourne, Victoria, Australia; *Andrew J. A. Holland*, The Children's Hospital at Westmead Clinical School, Faculty of Medicine and Health, University of Sydney, Sydney, New South Wales, Australia; *Stephen Honeybul*, Department of Neurosurgery, Sir Charles Gairdner Hospital, Perth, Western Australia, Australia; Department of Neurosurgery, Royal Perth Hospital, Perth, Western Australia, Australia; *Rosalind L. Jeffree*, Kenneth G. Jamieson Department of Neurosurgery, Royal Brisbane and Women's Hospital, Brisbane, Queensland, Australia; Royal Brisbane Clinical School, School of Medicine, University of Queensland, Brisbane, Queensland, Australia; *Chris Joyce*, Intensive Care Unit, Princess Alexandra Hospital, Brisbane, Queensland, Australia; School of Medicine, University of Queensland, Brisbane, Queensland, Australia; *Elizabeth Kendall*, Menzies Health Institute Queensland, Griffith University, Brisbane, Queensland, Australia; *Kate King*, John Hunter Trauma Service, John Hunter Hospital, Newcastle, New South Wales, Australia; College of Health, Medicine and Wellbeing, University of Newcastle, Newcastle, New South Wales, Australia; *Natasha A. Lannin*, Department of Neuroscience, Central Clinical School, Monash University, Melbourne, Victoria, Australia; Alfred Health, Melbourne, Victoria, Australia; *Meng Law,* Departments of Neuroscience and Radiology, Monash University, Melbourne, Victoria, Australia; Alfred Health, Melbourne, Victoria, Australia; Alzheimer's Disease Research Center & Department of Neurological Surgery, Keck School of Medicine, University of Southern California, Los Angeles, California, United States of America; *Andrew I. R. Maas,* Department of Neurosurgery, Antwerp University Hospital and University of Antwerp, Edegem, Belgium; *Adam Mahoney*, Trauma Service, Royal Hobart Hospital, Hobart, Tasmania, Australia; 2nd General Health Battalion, Australian Defence Force; *Peter Makin,* AUS-TBI Lived Experience Advisory Group; *Peter Mayhew,* AUS-TBI Lived Experience Advisory Group; *Alison McDonald,* AUS-TBI Lived Experience Advisory Group*; Skye McDonald*, School of Psychology, University of New South Wales, Sydney, New South Wales, Australia; *Stuart J. McDonald*, Department of Neuroscience, Central Clinical School, Monash University, Melbourne, Victoria, Australia; *Ancelin McKimmie*, School of Public Health and Preventive Medicine, Monash University, Melbourne, Victoria, Australia; *Robert McNamara,* Department of Intensive Care Medicine, Royal Perth Hospital, Perth, Western Australia, Australia; School of Medicine, Faculty of Health Sciences, Curtin University, Perth, Western Australia, Australia; *Shiv Meka,* Department of Health, Government of Western Australia, Perth, Western Australia, Australia; *David K. Menon*, Division of Anaesthesia, University of Cambridge, Addenbrooke's Hospital, Cambridge, United Kingdom; Wolfson Brain Imaging Centre, University of Cambridge, Cambridge, United Kingdom; *Gary Mitchell*, Emergency and Trauma Unit, Royal Brisbane and Women's Hospital, Brisbane, Queensland, Australia; Royal Brisbane Clinical Unit, University of Queensland, Brisbane, Queensland, Australia; Jamieson Trauma Institute, Brisbane, Queensland, Australia; Queensland Rugby Union; Brisbane, Queensland, Australia; *Rowena Mobbs*, Brain & Mind Centre, University of Sydney, Sydney, New South Wales, Australia; Macquarie University, Sydney, New South Wales, Australia; *Fatima A. Nasrallah,* Queensland Brain Institute, University of Queensland, Brisbane, Queensland, Australia; *Virginia F. J. Newcombe*, PACE Section, Department of Medicine, Addenbrooke's Hospital, University of Cambridge, Cambridge, United Kingdom; *Terence J. O'Brien*, Department of Neuroscience, Central Clinical School, Monash University, Melbourne, Victoria, Australia; *John H. Olver*, Epworth Healthcare, Melbourne, Victoria, Australia; Department of Medicine, Monash University, Melbourne, Victoria, Australia; *Gerard M. O'Reilly*, National Trauma Research Institute, Melbourne, Victoria, Australia; Emergency and Trauma Centre, The Alfred Hospital, Melbourne, Victoria, Australia; School of Public Health and Preventive Medicine, Monash University, Melbourne, Victoria, Australia; *Tamara Ownsworth*, The Hopkins Centre, Menzies Health Institute Queensland, Griffith University, Brisbane, Queensland, Australia; School of Applied Psychology, Griffith University, Brisbane, Queensland, Australia; Paul M. Parizel, University of Antwerp, Edegem, Belgium; Department of Radiology, Royal Perth Hospital & University of Western Australia, Perth, Western Australia, Australia; West Australian National Imaging Facility Node, Perth, Western Australia, Australia; *Michael Parr*, Intensive Care Unit, Liverpool Hospital, University of New South Wales, Sydney, New South Wales, Australia; Intensive Care Unit, Macquarie University Hospital, Macquarie University, Sydney, New South Wales, Australia; *Jennie L. Ponsford*, School of Psychological Sciences, Monash University, Melbourne, Victoria, Australia; Monash Epworth Rehabilitation Research Centre, Epworth Healthcare, Melbourne, Victoria, Australia; *Bruce Powell,* AUS-TBI Lived Experience Advisory Group; *Patricia Ratajczak,* AUS-TBI Lived Experience Advisory Group; *Danette Rause,* AUS-TBI Lived Experience Advisory Group; *Michael C. Reade*, Faculty of Medicine, University of Queensland, Royal Brisbane and Women's Hospital, Brisbane, Queensland, Australia; Joint Health Command, Australian Defence Force, Canberra, Australian Capital Territory, Australia; *Sandy Reeder*, Department of Neuroscience, Central Clinical School, Monash University, Melbourne, Victoria, Australia; Department of Epidemiology and Preventive Medicine, School of Public Health and Preventive Medicine, Monash University, Melbourne, Victoria, Australia; *Christopher Reid*, School of Public Health, Faculty of Health Sciences, Curtin University, Perth, Western Australia, Australia; Department of Epidemiology and Preventive Medicine, School of Public Health and Preventive Medicine, Monash University, Melbourne, Victoria, Australia; *Julia Robertson,* AUS-TBI Lived Experience Advisory Group; *Suzanne Robinson*, School of Population Health, Faculty of Health Sciences, Curtin University, Perth Western Australia, Australia; *Stephen E. Rose*, The Australian e-Health Research Centre, Commonwealth Scientific and Industrial Research Organisation, Brisbane, Queensland, Australia; *Jeffrey V. Rosenfeld*, Department of Neurosurgery, The Alfred Hospital, Melbourne, Victoria, Australia; Department of Surgery, Monash University, Melbourne, Victoria, Australia; F. Edward Hébert School of Medicine, Uniformed Services University of The Health Sciences, Bethesda, Maryland, United States of America; *Jason P. Ross*, Molecular Diagnostic Solutions, Health and Biosecurity, Commonwealth Scientific and Industrial Research Organisation, Australia; *Nick Rushworth*, Brain Injury Australia, Sydney, New South Wales, Australia; *Adam Scheinberg*, Neurodevelopment and Rehabilitation Research, Murdoch Children's Research Institute, Melbourne, Victoria, Australia; Department of Paediatrics, University of Melbourne, Melbourne, Victoria, Australia; *Bridgette D. Semple*, Department of Neuroscience, Central Clinical School, Monash University, Melbourne, Victoria, Australia; Alfred Health, Melbourne, Victoria, Australia; Department of Medicine, Royal Melbourne Hospital, The University of Melbourne, Melbourne, Victoria, Australia; *Sandy R. Shultz*, Department of Neuroscience, Central Clinical School, Monash University, Melbourne, Victoria, Australia; *Grahame K. Simpson,* Brain Injury Rehabilitation Research Group, Ingham Institute for Applied Medical Research, Sydney, New South Wales, Australia; John Walsh Centre for Rehabilitation Research, Sydney School of Medicine, University of Sydney, Sydney, New South Wales, Australia; *Warwick J. Teague*, Trauma Service & Department of Paediatric Surgery, The Royal Children's Hospital, Melbourne, Victoria, Australia; Surgical Research, Murdoch Children's Research Institute, Melbourne, Victoria, Australia; Department of Paediatrics, University of Melbourne, Melbourne, Victoria, Australia; *Leanne Togher*, Speech Pathology, School of Health Sciences, Faculty of Medicine and Health, University of Sydney, Sydney, New South Wales, Australia; *Andrew A. Udy*, Australian and New Zealand Intensive Care Research Centre, School of Public Health and Preventive Medicine, Monash University, Melbourne, Victoria, Australia; Department of Intensive Care and Hyperbaric Medicine, The Alfred Hospital, Melbourne, Victoria, Australia; *Kirsten Vallmuur*, Centre for Healthcare Transformation, Australian Centre for Health Services Innovation, Queensland University of Technology, Brisbane, Queensland, Australia; Jamieson Trauma Institute, Brisbane, Queensland, Australia; *Dinesh Varma*, Department of Radiology, The Alfred Hospital, Melbourne, Victoria, Australia; Department of Surgery, Monash University, Melbourne, Victoria, Australia; National Trauma Research Institute, Melbourne, Victoria, Australia; *James Vickers*, Wicking Dementia Research and Education Centre, College of Health and Medicine, University of Tasmania, Hobart, Tasmania, Australia; *Janet Wagland*, Brightwater Group, Perth, Western Australia, Australia; *James Walsham*, Intensive Care Unit, Princess Alexandra Hospital, Brisbane, Queensland, Australia; School of Medicine, University of Queensland, Brisbane, Queensland, Australia; *Adam J. Wells*, Department of Neurosurgery, Adelaide Hospital, Adelaide, South Australia, Australia; Department of Surgery, University of Adelaide, Adelaide, South Australia, Australia; Neurosurgical Research Foundation, Adelaide, South Australia, Australia; *Luke Whiley*, Health Futures Institute, Murdoch University, Perth, Western Australia, Australia; Perron Institute for Neurological and Translational Science, Perth, Western Australia, Australia; *Gavin Williams*, Department of Physiotherapy, Epworth Healthcare, Melbourne, Victoria, Australia; Department of Physiotherapy, University of Melbourne, Melbourne, Victoria, Australia; *Jodie K. Williams*, National Critical Care and Trauma Response Centre, Royal Darwin Hospital, Darwin, Northern Territory, Australia; *Roslind Witham*, AUS-TBI Lived Experience Advisory Group; *David K. Wright*, Department of Neuroscience, Central Clinical School, Monash University, Melbourne, Victoria, Australia; *Louise York*, Australian Institute of Health and Welfare, Canberra, Australian Capital Territory, Australia; *Jesse T. Young*, Centre for Health Equity, Melbourne School of Population and Global Health, The University of Melbourne, Melbourne, Victoria, Australia; Centre for Adolescent Health, Murdoch Children's Research Institute, Melbourne, Victoria, Australia; School of Population and Global Health, University of Western Australia, Perth, Western Australia, Australia; National Drug Research Institute, Curtin University, Perth, Western Australia, Australia; *Heidi Zeeman*, The Hopkins Centre, Menzies Health Institute Queensland, Griffith University, Brisbane, Queensland, Australia.

## Abbreviations Used

ADL: activities of daily living
AUS-TBI: The Australian Traumatic Brain Injury Initiative
CADTH: Canadian Agency for Drugs and Technologies in Health
CINAHL: Cumulated Index to Nursing and Allied Health Literature
CT: computed tomography
EMTREE: Excerpta Medica Tree
EQ5D: EuroQol-5 Dimensions
FIM: Functional Independence Measure
GOSE-E: Glasgow Outcome Scale-Extended
GOS-E PEDS: Glasgow Outcome Scale Modified for Children
ICF: International Classification of Functional Disability and Health
MeSH: Medical Subject Headings
MPAI: Mayo-Portland Adaptability Inventory
PEDS-QL: Paediatric Quality of Life Scale
PRISMA: Preferred Reporting Items for Systematic Reviews and Meta-Analyses
PROSPERO: Prospective Register of Systematic Reviews
QoL: quality of life
RTS: return to study
RTW: return to work
SDQ: Strengths and Difficulties Questionnaire
SWLS: Satisfaction With Life Scale (adult)
WEEFIM: Functional Independence Measure for Children
WHO: World Health Organization
