# Supplementary material for: The Australian Traumatic Brain Injury Initiative: Review and Recommendations for Outcome Measures for Use With Adults and Children After Moderate-to-Severe Traumatic Brain Injury
Source: Neurotrauma Rep. 2024 Apr 11;5(1):387–408. doi: 10.1089/neur.2023.0127 (PMC11035854; doi:10.1089/neur.2023.0127)
Supplement: Supplemental data [file Suppl_Text.docx]

Supplementary Material

In accompaniment of Ponsford, Hicks et al. (2023, submitted) “The Australian Traumatic Brain Injury Initiative: Review and recommendations for outcome measures for use with children and adults following moderate-to-severe traumatic brain injury”.

Comprising:

1. Meta-data and search strategies (this document)

2. Paper 7_AUS-TBI_Supplementary file 2_Supplementary Tables.docx

3. Paper 7_AUS-TBI_Supplementary file 3_Investigators names and affiliations.xlsx

**Search Strategies**

**A. CINAHL via EBSCOhost**

S5 S3 AND S4 284

S4 S1 AND S2 4,221

S3 (TI (systematic* N3 review*)) OR (AB (systematic* N3 review*)) OR (TI (systematic* N3 bibliographic*)) OR (AB (systematic* N3 bibliographic*)) OR (TI (systematic* N3 literature)) OR (AB (systematic* N3 literature)) OR (TI (comprehensive* N3 literature)) OR (AB (comprehensive* N3 literature)) OR (TI (comprehensive* N3 bibliographic*)) OR (AB (comprehensive* N3 bibliographic*)) OR (TI (integrative N3 review)) OR (AB (integrative N3 review)) OR (JN "Cochrane Database of Systematic Reviews") OR (TI (information N2 synthesis)) OR (TI (data N2 synthesis)) OR (AB (information N2 synthesis)) OR (AB (data N2 synthesis)) OR (TI (data N2 extract*)) OR (AB (data N2 extract*)) OR (TI (medline OR pubmed OR psyclit OR cinahl OR (psycinfo not "psycinfo database") OR "web of science" OR scopus OR embase)) OR (AB (medline OR pubmed OR psyclit OR cinahl OR (psycinfo not "psycinfo database") OR "web of science" OR scopus OR embase)) OR (MH "Systematic Review") OR (MH "Meta Analysis") OR (TI (meta-analy* OR metaanaly*)) OR (AB (meta-analy* OR metaanaly*)) 253,729

S2 ( (MH "Activities of Daily Living+") OR (MH "Functional Status") OR (MH "Quality of Life+") OR (MH "Treatment Outcome") OR (MH "Outcome Assessment") OR (MH "Advanced Trauma Life Support Care") OR (MH "Life Support Care") OR (MH "Long Term Care") OR (MH "Survival") OR (MH "Mortality") OR (MH "Survival Analysis+") OR (MH "Fatal Outcome") OR (MH "Hospital Mortality") OR (MH "Patient-Reported Outcomes") OR (MH "Disability Evaluation+") OR (MH "International Classification of Functioning, Disability, and Health") OR (MH "Disability-Adjusted Life Years") OR (MH "Quality-Adjusted Life Years") OR (MH "Job Re-Entry") OR (MH "School Re-Entry") OR (MH "Patient Satisfaction") OR (MH "Personal Satisfaction") OR (MH "Morbidity") OR (MH "Rehabilitation, Vocational+") OR (MH "Employment") OR (MH "Employment, Supported") OR (MH "Unemployment") OR (MH "Social Participation") OR (MH "Work Engagement") ) OR TI ( Activities of Daily Living OR ADL OR Daily Living Activities OR Daily Living Activity OR Chronic Limitation of Activity OR Independent Living OR "Aging in Place" OR Self-Management OR "Quality of Life" OR Life Quality OR Instrumental OR Life Support OR "Prolongation of Life" OR Life Prolongation OR Extraordinary Treatment* OR Longitudinal OR Long-Term OR Longterm OR Survival* OR Mortalit* OR Fatalit* OR Death* OR Cox Model* OR Hazard Model* OR Hazards Model* OR Kaplan-Meier OR Disabilit* OR Work* OR Vocation* OR DALY* OR QALY* OR QUALY* OR Quality Adjusted Life Year* OR Healthy Years Equivalent* OR Adjusted Life Year* OR Satisfaction OR Morbidit* OR Productiv* OR Employment OR Occupational Status OR Unemployment OR Participation OR Engagement OR Function* OR Outcome* OR Care OR "Back to School" OR ((Return* OR Resum*) N1 (School* OR Education OR Study)) OR ((School* OR Education OR Study) N1 (Return* OR Resum*)) ) OR AB ( Activities of Daily Living OR ADL OR Daily Living Activities OR Daily Living Activity OR Chronic Limitation of Activity OR Independent Living OR "Aging in Place" OR Self-Management OR "Quality of Life" OR Life Quality OR Instrumental OR Life Support OR "Prolongation of Life" OR Life Prolongation OR Extraordinary Treatment* OR Longitudinal OR Long-Term OR Longterm OR Survival* OR Mortalit* OR Fatalit* OR Death* OR Cox Model* OR Hazard Model* OR Hazards Model* OR Kaplan-Meier OR Disabilit* OR Work* OR Vocation* OR DALY* OR QALY* OR QUALY* OR Quality Adjusted Life Year* OR Healthy Years Equivalent* OR Adjusted Life Year* OR Satisfaction OR Morbidit* OR Productiv* OR Employment OR Occupational Status OR Unemployment OR Participation OR Engagement OR Function* OR Outcome* OR Care OR "Back to School" OR ((Return* OR Resum*) N1 (School* OR Education OR Study)) OR ((School* OR Education OR Study) N1 (Return* OR Resum*)) ) 375,704

S1 ( ((MH "Intracranial Hemorrhage") OR (MH "Cerebral Hemorrhage") OR (MH "Brain Injuries") OR (MH "Brain Contusions") OR (MH "Head Injuries")) AND (MH "Trauma+") ) OR TI ( (Brain* N1 (Injur* OR Trauma* OR Posttrauma*)) OR ((Posttrauma* OR Trauma* OR Injur*) N1 Brain*) ) OR AB ( (Brain* N1 (Injur* OR Trauma* OR Posttrauma*)) OR ((Posttrauma* OR Trauma* OR Injur*) N1 Brain*) ) 29,792

**B. Cochrane Database of Systematic Reviews**

([mh "Cerebral Hemorrhage, Traumatic"] OR [mh "Brain Injuries, Diffuse"] OR [mh "Diffuse Axonal Injury"] OR [mh "Brain Injuries, Traumatic"] OR [mh "Brain Contusion"] OR [mh "Head Injuries, Closed"] OR [mh "Head Injuries, Penetrating"] OR ((Brain* NEAR (Injur* OR Trauma* OR Posttrauma*)) OR ((Posttrauma* OR Trauma* OR Injur*) NEAR Brain*)):ti,ab) AND ([mh "Activities of Daily Living"] OR [mh "Functional Status"] OR [mh "Independent Living"] OR [mh "Quality of Life"] OR [mh "Treatment Outcome"] OR [mh "Outcome Assessment, Health Care"] OR [mh "Outcome and Process Assessment, Health Care"] OR [mh "Advanced Trauma Life Support Care"] OR [mh "Life Support Care"] OR [mh "Longitudinal Studies"] OR [mh "Long-Term Care"] OR [mh Survival] OR [mh Mortality] OR [mh "Survival Rate"] OR [mh "Survival Analysis"] OR [mh "Fatal Outcome"] OR [mh "Hospital Mortality"] OR [mh "Patient Outcome Assessment"] OR [mh "Patient Reported Outcome Measures"] OR [mh "Disability Evaluation"] OR [mh "International Classification of Functioning, Disability and Health"] OR [mh "Disability-Adjusted Life Years"] OR [mh "Quality-Adjusted Life Years"] OR [mh "Return to Work"] OR [mh "Return to School"] OR [mh "Patient Satisfaction"] OR [mh "Personal Satisfaction"] OR [mh "Morbidity"] OR [mh "Rehabilitation, Vocational"] OR [mh "Employment"] OR [mh "Employment, Supported"] OR [mh "Unemployment"] OR [mh "Social Participation"] OR [mh "Community Participation"] OR [mh "Work Engagement"] OR [mh /MO] OR (Activities of Daily Living OR ADL OR Daily Living Activities OR Daily Living Activity OR Chronic Limitation of Activity OR Independent Living OR "Aging in Place" OR Self-Management OR "Quality of Life" OR Life Quality OR Instrumental OR Life Support OR "Prolongation of Life" OR Life Prolongation OR Extraordinary Treatment* OR Longitudinal OR Long-Term OR Longterm OR Survival* OR Mortalit* OR Fatalit* OR Death* OR Cox Model* OR Hazard Model* OR Hazards Model* OR Kaplan-Meier OR Disabilit* OR Work* OR Vocation* OR DALY* OR QALY* OR QUALY* OR Quality Adjusted Life Year* OR Healthy Years Equivalent* OR Adjusted Life Year* OR Satisfaction OR Morbidit* OR Productiv* OR Employment OR Occupational Status OR Unemployment OR Participation OR Engagement OR Function* OR Outcome* OR Care OR "Back to School" OR ((Return* OR Resum*) NEAR (School* OR Education OR Study)) OR ((School* OR Education OR Study) NEAR (Return* OR Resum*))):ti,ab) in Cochrane Reviews, Cochrane Protocols 117

**C. Embase 1974 to 2022 Week 13 via Ovid SP**

1 Diffuse Brain Injury/ OR Diffuse Axonal Injury/ OR exp Traumatic Brain Injury/ OR Brain Contusion/ OR Head Injury/ OR (Brain* adj1 (Injur* OR Trauma* OR Posttrauma*)).ti,ab. OR ((Posttrauma* OR Trauma* OR Injur*) adj1 Brain*).ti,ab. (165162)

2 Daily Living Activity/ OR Functional Status/ OR Independent Living/ OR exp "Quality of Life"/ OR exp "Quality of Life Assessment"/ OR Treatment Outcome/ OR Outcome Assessment/ OR Advanced Trauma Life Support/ OR Long Term Care/ OR Longitudinal Study/ OR Survival/ OR Mortality/ OR Mortality Rate/ OR Mortality Risk/ OR Mortality Risk Score/ OR Standardized Mortality Ratio/ OR Survival Rate/ OR exp Survival Analysis/ OR Fatality/ OR exp Hospital Mortality/ OR "All Cause Mortality"/ OR Patient-Reported Outcome/ OR "International Classification of Functioning, Disability and Health"/ OR Disability-Adjusted Life Year/ OR Quality Adjusted Life Year/ OR "Return to Work"/ OR "Return to School"/ OR Patient Satisfaction/ OR Life Satisfaction/ OR Morbidity/ OR Vocational Rehabilitation/ OR exp Employment/ OR Social Participation/ OR exp Community Participation/ OR exp Work Engagement/ OR (Activities of Daily Living OR ADL OR Daily Living Activities OR Daily Living Activity OR Chronic Limitation of Activity OR Independent Living OR "Aging in Place" OR Self-Management OR "Quality of Life" OR Life Quality OR Instrumental OR Life Support OR "Prolongation of Life" OR Life Prolongation OR Extraordinary Treatment* OR Longitudinal OR Long-Term OR Longterm OR Survival* OR Mortalit* OR Fatalit* OR Death* OR Cox Model* OR Hazard Model* OR Hazards Model* OR Kaplan-Meier OR Disabilit* OR Work* OR Vocation* OR DALY* OR QALY* OR QUALY* OR Quality Adjusted Life Year* OR Healthy Years Equivalent* OR Adjusted Life Year* OR Satisfaction OR Morbidit* OR Productiv* OR Employment OR Occupational Status OR Unemployment OR Participation OR Engagement OR Function* OR Outcome* OR Care OR "Back to School" OR ((Return* OR Resum*) adj1 (School* OR Education OR Study)) OR ((School* OR Education OR Study) adj1 (Return* OR Resum*))).ti,ab. (14584316)

3 (systematic review OR meta-analysis).pt. OR meta-analysis/ OR systematic review/ OR systematic reviews as topic/ OR meta-analysis as topic/ OR "meta analysis (topic)"/ OR "systematic review (topic)"/ OR exp technology assessment, biomedical/ OR network meta-analysis/ OR ((systematic* adj3 (review* OR overview*)) OR (methodologic* adj3 (review* OR overview*))).ti,ab,kf,kw. OR ((quantitative adj3 (review* OR overview* OR synthes*)) OR (research adj3 (integrati* OR overview*))).ti,ab,kf,kw. OR ((integrative adj3 (review* OR overview*)) OR (collaborative adj3 (review* OR overview*)) OR (pool* adj3 analy*)).ti,ab,kf,kw. OR (data synthes* OR data extraction* OR data abstraction*).ti,ab,kf,kw. OR (handsearch* OR hand search*).ti,ab,kf,kw. OR (mantel haenszel OR peto OR der simonian OR dersimonian OR fixed effect* OR latin square*).ti,ab,kf,kw. OR (met analy* OR metanaly* OR technology assessment* OR HTA OR HTAs OR technology overview* OR technology appraisal*).ti,ab,kf,kw. OR (meta regression* OR metaregression*).ti,ab,kf,kw. OR (meta-analy* OR metaanaly* OR systematic review* OR biomedical technology assessment* OR bio-medical technology assessment*).mp,hw. OR (medline OR cochrane OR pubmed OR medlars OR embase OR cinahl).ti,ab,hw. OR (cochrane OR (health adj2 technology assessment) OR evidence report).jw. OR (comparative adj3 (efficacy OR effectiveness)).ti,ab,kf,kw. OR (outcomes research OR relative effectiveness).ti,ab,kf,kw. OR ((indirect OR indirect treatment OR mixed-treatment OR bayesian) adj3 comparison*).ti,ab,kf,kw. OR (multi* adj3 treatment adj3 comparison*).ti,ab,kf,kw. OR (mixed adj3 treatment adj3 (meta-analy* OR metaanaly*)).ti,ab,kf,kw. OR umbrella review*.ti,ab,kf,kw. OR (multi* adj2 paramet* adj2 evidence adj2 synthesis).ti,ab,kw,kf. OR (multiparamet* adj2 evidence adj2 synthesis).ti,ab,kw,kf. OR (multi-paramet* adj2 evidence adj2 synthesis).ti,ab,kw,kf. (854331)

4 and/1-3 (5215)

5 letter.pt. OR editorial.pt. OR (animal/ not (animal/ and human/)) {Including Related Terms} (42646)

6 4 not 5 (5212)

7 limit 6 to english language (5079)

8 limit 7 to medline (651)

9 7 not 8 (4428)

10 limit 9 to embase (3459)

**D. Epistemonikos**

Brain* AND (Injur* OR Trauma* OR Posttrauma*)

Category: Broad Syntheses (98), Structured Summaries (152), Systematic Reviews: Cochrane review [No] (3,038)

**E. Ovid MEDLINE(R) ALL <1946 to April 01, 2022>**

1 Cerebral Hemorrhage, Traumatic/ OR Brain Injuries, Diffuse/ OR Diffuse Axonal Injury/ OR Brain Injuries, Traumatic/ OR Brain Contusion/ OR Head Injuries, Closed/ OR Head Injuries, Penetrating/ OR (Brain* adj1 (Injur* OR Trauma* OR Posttrauma*)).ti,ab. OR ((Posttrauma* OR Trauma* OR Injur*) adj1 Brain*).ti,ab. (82686)

2 exp Activities of Daily Living/ OR Functional Status/ OR Independent Living/ OR "Quality of Life"/ OR Treatment Outcome/ OR Outcome Assessment, Health Care/ OR "Outcome and Process Assessment, Health Care"/ OR Advanced Trauma Life Support Care/ OR Life Support Care/ OR Longitudinal Studies/ OR Long-Term Care/ OR Survival/ OR Mortality/ OR Survival Rate/ OR exp Survival Analysis/ OR Fatal Outcome/ OR Hospital Mortality/ OR Patient Outcome Assessment/ OR Patient Reported Outcome Measures/ OR exp Disability Evaluation/ OR "International Classification of Functioning, Disability and Health"/ OR Disability-Adjusted Life Years/ OR Quality-Adjusted Life Years/ OR "Return to Work"/ OR "Return to School"/ OR Patient Satisfaction/ OR Personal Satisfaction/ OR Morbidity/ OR Rehabilitation, Vocational/ OR Employment/ OR Employment, Supported/ OR Unemployment/ OR Social Participation/ OR Community Participation/ OR Work Engagement/ OR Mortality.fs. OR (Activities of Daily Living OR ADL OR Daily Living Activities OR Daily Living Activity OR Chronic Limitation of Activity OR Independent Living OR "Aging in Place" OR Self-Management OR "Quality of Life" OR Life Quality OR Instrumental OR Life Support OR "Prolongation of Life" OR Life Prolongation OR Extraordinary Treatment* OR Longitudinal OR Long-Term OR Longterm OR Survival* OR Mortalit* OR Fatalit* OR Death* OR Cox Model* OR Hazard Model* OR Hazards Model* OR Kaplan-Meier OR Disabilit* OR Work* OR Vocation* OR DALY* OR QALY* OR QUALY* OR Quality Adjusted Life Year* OR Healthy Years Equivalent* OR Adjusted Life Year* OR Satisfaction OR Morbidit* OR Productiv* OR Employment OR Occupational Status OR Unemployment OR Participation OR Engagement OR Function* OR Outcome* OR Care OR "Back to School" OR ((Return* OR Resum*) adj1 (School* OR Education OR Study)) OR ((School* OR Education OR Study) adj1 (Return* OR Resum*))).ti,ab. (11285625)

3 (systematic review OR meta-analysis).pt. OR meta-analysis/ OR systematic review/ OR systematic reviews as topic/ OR meta-analysis as topic/ OR "meta analysis (topic)"/ OR "systematic review (topic)"/ OR exp technology assessment, biomedical/ OR network meta-analysis/ OR ((systematic* adj3 (review* OR overview*)) OR (methodologic* adj3 (review* OR overview*))).ti,ab,kf,kw. OR ((quantitative adj3 (review* OR overview* OR synthes*)) OR (research adj3 (integrati* OR overview*))).ti,ab,kf,kw. OR ((integrative adj3 (review* OR overview*)) OR (collaborative adj3 (review* OR overview*)) OR (pool* adj3 analy*)).ti,ab,kf,kw. OR (data synthes* OR data extraction* OR data abstraction*).ti,ab,kf,kw. OR (handsearch* OR hand search*).ti,ab,kf,kw. OR (mantel haenszel OR peto OR der simonian OR dersimonian OR fixed effect* OR latin square*).ti,ab,kf,kw. OR (met analy* OR metanaly* OR technology assessment* OR HTA OR HTAs OR technology overview* OR technology appraisal*).ti,ab,kf,kw. OR (meta regression* OR metaregression*).ti,ab,kf,kw. OR (meta-analy* OR metaanaly* OR systematic review* OR biomedical technology assessment* OR bio-medical technology assessment*).mp,hw. OR (medline OR cochrane OR pubmed OR medlars OR embase OR cinahl).ti,ab,hw. OR (cochrane OR (health adj2 technology assessment) OR evidence report).jw. OR (comparative adj3 (efficacy OR effectiveness)).ti,ab,kf,kw. OR (outcomes research OR relative effectiveness).ti,ab,kf,kw. OR ((indirect OR indirect treatment OR mixed-treatment OR bayesian) adj3 comparison*).ti,ab,kf,kw. OR (multi* adj3 treatment adj3 comparison*).ti,ab,kf,kw. OR (mixed adj3 treatment adj3 (meta-analy* OR metaanaly*)).ti,ab,kf,kw. OR umbrella review*.ti,ab,kf,kw. OR (multi* adj2 paramet* adj2 evidence adj2 synthesis).ti,ab,kw,kf. OR (multiparamet* adj2 evidence adj2 synthesis).ti,ab,kw,kf. OR (multi-paramet* adj2 evidence adj2 synthesis).ti,ab,kw,kf. (591615)

4 and/1-3 (2763)

5 Comment/ OR Letter/ OR Editorial/ OR (animal/ not (animal/ and human/)) {Including Related Terms} (39039)

6 4 not 5 (2760)

7 limit 6 to english language (2696)

**F. APA PsycInfo <1806 to March Week 4 2022>**

1 (Cerebral Hemorrhage/ and Trauma/) OR Traumatic Brain Injury/ OR Head Injuries/ OR (Brain* adj1 (Injur* OR Trauma* OR Posttrauma*)).ti,ab. OR ((Posttrauma* OR Trauma* OR Injur*) adj1 Brain*).ti,ab. (34910)

2 "Activities of Daily Living"/ OR Functional Status/ OR exp "Quality of Life"/ OR Treatment Outcomes/ OR Longitudinal Studies/ OR Long Term Care/ OR Patient Reported Outcome Measures/ OR exp "Treatment Process and Outcome Measures"/ OR Life Sustaining Treatment/ OR Disability Evaluation/ OR Reemployment/ OR Life Satisfaction/ OR Satisfaction/ OR Morbidity/ OR Vocational Rehabilitation/ OR Supported Employment/ OR Unemployment/ OR (Activities of Daily Living OR ADL OR Daily Living Activities OR Daily Living Activity OR Chronic Limitation of Activity OR Independent Living OR "Aging in Place" OR Self-Management OR "Quality of Life" OR Life Quality OR Instrumental OR Life Support OR "Prolongation of Life" OR Life Prolongation OR Extraordinary Treatment* OR Longitudinal OR Long-Term OR Longterm OR Survival* OR Mortalit* OR Fatalit* OR Death* OR Cox Model* OR Hazard Model* OR Hazards Model* OR Kaplan-Meier OR Disabilit* OR Work* OR Vocation* OR DALY* OR QALY* OR QUALY* OR Quality Adjusted Life Year* OR Healthy Years Equivalent* OR Adjusted Life Year* OR Satisfaction OR Morbidit* OR Productiv* OR Employment OR Occupational Status OR Unemployment OR Participation OR Engagement OR Function* OR Outcome* OR Care OR "Back to School" OR ((Return* OR Resum*) adj1 (School* OR Education OR Study)) OR ((School* OR Education OR Study) adj1 (Return* OR Resum*))).ti,ab. (2378149)

3 (systematic review OR meta-analysis).pt. OR meta-analysis/ OR systematic review/ OR systematic reviews as topic/ OR meta-analysis as topic/ OR "meta analysis (topic)"/ OR "systematic review (topic)"/ OR network meta-analysis/ OR ((systematic* adj3 (review* OR overview*)) OR (methodologic* adj3 (review* OR overview*))).tw,ot. OR ((quantitative adj3 (review* OR overview* OR synthes*)) OR (research adj3 (integrati* OR overview*))).tw,ot. OR ((integrative adj3 (review* OR overview*)) OR (collaborative adj3 (review* OR overview*)) OR (pool* adj3 analy*)).tw,ot. OR (data synthes* OR data extraction* OR data abstraction*).tw,ot. OR (handsearch* OR hand search*).tw,ot. OR (mantel haenszel OR peto OR der simonian OR dersimonian OR fixed effect* OR latin square*).tw,ot. OR (met analy* OR metanaly* OR technology assessment* OR HTA OR HTAs OR technology overview* OR technology appraisal*).tw,ot. OR (meta regression* OR metaregression*).tw,ot. OR (meta-analy* OR metaanaly* OR systematic review* OR biomedical technology assessment* OR bio-medical technology assessment*).mp,hw. OR (medline OR cochrane OR pubmed OR medlars OR embase OR cinahl).ti,ab,hw. OR (cochrane OR (health adj2 technology assessment) OR evidence report).jw. OR (comparative adj3 (efficacy OR effectiveness)).tw,ot. OR (outcomes research OR relative effectiveness).tw,ot. OR ((indirect OR indirect treatment OR mixed-treatment OR bayesian) adj3 comparison*).tw,ot. OR (multi* adj3 treatment adj3 comparison*).tw,ot. OR (mixed adj3 treatment adj3 (meta-analy* OR metaanaly*)).tw,ot. OR umbrella review*.tw,ot. OR (multi* adj2 paramet* adj2 evidence adj2 synthesis).tw,ot. OR (multiparamet* adj2 evidence adj2 synthesis).tw,ot. OR (multi-paramet* adj2 evidence adj2 synthesis).tw,ot. (115558)

4 and/1-3 (1077)

5 limit 4 to english language (1034)

**G. PubMed (Excluding MEDLINE)**

("Cerebral Hemorrhage, Traumatic"[MH] OR "Brain Injuries, Diffuse"[MH] OR "Diffuse Axonal Injury"[MH] OR "Brain Injuries, Traumatic"[MH] OR "Brain Contusion"[MH] OR "Head Injuries, Closed"[MH] OR "Head Injuries, Penetrating"[MH] OR (Brain*[TIAB] AND (Injur*[TIAB] OR Trauma*[TIAB] OR Posttrauma*[TIAB]))) AND ("Activities of Daily Living"[MH] OR "Functional Status"[MH] OR "Independent Living"[MH] OR "Quality of Life"[MH] OR "Treatment Outcome"[MH] OR "Outcome Assessment, Health Care"[MH] OR "Outcome and Process Assessment, Health Care"[MH] OR "Advanced Trauma Life Support Care"[MH] OR "Life Support Care"[MH] OR "Longitudinal Studies"[MH] OR "Long-Term Care"[MH] OR Survival[MH] OR Mortality[MH] OR "Survival Rate"[MH] OR "Survival Analysis"[MH] OR "Fatal Outcome"[MH] OR "Hospital Mortality"[MH] OR "Patient Outcome Assessment"[MH] OR "Patient Reported Outcome Measures"[MH] OR "Disability Evaluation"[MH] OR "International Classification of Functioning, Disability and Health"[MH] OR "Disability-Adjusted Life Years"[MH] OR "Quality-Adjusted Life Years"[MH] OR "Return to Work"[MH] OR "Return to School"[MH] OR "Patient Satisfaction"[MH] OR "Personal Satisfaction"[MH] OR "Morbidity"[MH] OR "Rehabilitation, Vocational"[MH] OR "Employment"[MH] OR "Employment, Supported"[MH] OR "Unemployment"[MH] OR "Social Participation"[MH] OR "Community Participation"[MH] OR "Work Engagement"[MH] OR Mortality[SH] OR "Activities of Daily Living"[TIAB] OR ADL[TIAB] OR "Daily Living Activities"[TIAB] OR "Daily Living Activity"[TIAB] OR "Chronic Limitation of Activity"[TIAB] OR "Independent Living"[TIAB] OR "Aging in Place"[TIAB] OR Self-Management[TIAB] OR "Quality of Life"[TIAB] OR "Life Quality"[TIAB] OR Instrumental[TIAB] OR "Life Support"[TIAB] OR "Prolongation of Life"[TIAB] OR "Life Prolongation"[TIAB] OR Extraordinary Treatment*[TIAB] OR Longitudinal[TIAB] OR Long-Term[TIAB] OR Longterm[TIAB] OR Survival*[TIAB] OR Mortalit*[TIAB] OR Fatalit*[TIAB] OR Death*[TIAB] OR Cox Model*[TIAB] OR Hazard Model*[TIAB] OR Hazards Model*[TIAB] OR Kaplan-Meier[TIAB] OR Disabilit*[TIAB] OR Work*[TIAB] OR Vocation*[TIAB] OR DALY*[TIAB] OR QALY*[TIAB] OR QUALY*[TIAB] OR Quality Adjusted Life Year*[TIAB] OR Healthy Years Equivalent*[TIAB] OR Adjusted Life Year*[TIAB] OR Satisfaction[TIAB] OR Morbidit*[TIAB] OR Productiv*[TIAB] OR Employment[TIAB] OR Occupational Status[TIAB] OR Unemployment[TIAB] OR Participation[TIAB] OR Engagement[TIAB] OR Function*[TIAB] OR Outcome*[TIAB] OR Care[TIAB] OR "Back to School"[TIAB] OR ((Return*[TIAB] OR Resum*[TIAB]) AND (School*[TIAB] OR Education[TIAB] OR Study[TIAB]))) AND ("Systematic"[Filter] OR "meta-analysis"[pt] OR "meta-analysis as topic"[mh] OR meta analy*[tw] OR metanaly*[tw] OR metaanaly*[tw] OR met analy*[tw] OR integrative research[tiab] OR integrative review*[tiab] OR integrative overview*[tiab] OR research integration*[tiab] OR research overview*[tiab] OR collaborative review*[tiab] OR collaborative overview*[tiab] OR "systematic review"[pt] OR "systematic reviews as topic"[mh] OR systematic review*[tiab] OR technology assessment*[tiab] OR technology overview*[tiab] OR technology appraisal*[tiab] OR "Technology Assessment, Biomedical"[mh] OR HTA[tiab] OR HTAs[tiab] OR comparative efficacy[tiab] OR comparative effectiveness[tiab] OR outcomes research[tiab] OR indirect comparison*[tiab] OR Bayesian comparison[tiab] OR ((indirect treatment[tiab] OR mixed-treatment[tiab]) AND comparison*[tiab]) OR Embase*[tiab] OR Cinahl*[tiab] OR systematic overview*[tiab] OR methodological overview*[tiab] OR methodologic overview*[tiab] OR methodological review*[tiab] OR methodologic review*[tiab] OR quantitative review*[tiab] OR quantitative overview*[tiab] OR quantitative synthes*[tiab] OR pooled analy*[tiab] OR Cochrane[tiab] OR Medline[tiab] OR Pubmed[tiab] OR Medlars[tiab] OR handsearch*[tiab] OR hand search*[tiab] OR meta-regression*[tiab] OR metaregression*[tiab] OR data synthes*[tiab] OR data extraction[tiab] OR data abstraction*[tiab] OR mantel haenszel[tiab] OR peto[tiab] OR der-simonian[tiab] OR dersimonian[tiab] OR fixed effect*[tiab] OR multiple treatment comparison[tiab] OR mixed treatment meta-analys*[tiab] OR umbrella review*[tiab] OR ((multiple paramet*[tiab]) AND (evidence synthesis[tiab])) OR ((multi-paramet*[tiab]) AND (evidence synthesis[tiab])) OR ((multiparameter*[tiab]) AND (evidence synthesis[tiab])) OR "Cochrane Database Syst Rev"[Journal] OR "health technology assessment winchester, england"[Journal] OR "Evid Rep Technol Assess (Full Rep)"[Journal] OR "Evid Rep Technol Assess (Summ)"[Journal] OR "Int J Technol Assess Health Care"[Journal] OR "GMS Health Technol Assess"[Journal] OR "Health Technol Assess (Rockv)"[Journal] OR "Health Technol Assess Rep"[Journal]) AND English[LA] NOT MEDLINE[SB] 605

**H. SPORTDiscus via EBSCOhost**

S4 S1 AND S2 AND S3 Limiters - Language: English 305

S3 (TI (systematic* N3 review*)) OR (AB (systematic* N3 review*)) OR (TI (systematic* N3 bibliographic*)) OR (AB (systematic* N3 bibliographic*)) OR (TI (systematic* N3 literature)) OR (AB (systematic* N3 literature)) OR (TI (comprehensive* N3 literature)) OR (AB (comprehensive* N3 literature)) OR (TI (comprehensive* N3 bibliographic*)) OR (AB (comprehensive* N3 bibliographic*)) OR (TI (integrative N3 review)) OR (AB (integrative N3 review)) OR (JN "Cochrane Database of Systematic Reviews") OR (TI (information N2 synthesis)) OR (TI (data N2 synthesis)) OR (AB (information N2 synthesis)) OR (AB (data N2 synthesis)) OR (TI (data N2 extract*)) OR (AB (data N2 extract*)) OR (TI (medline OR pubmed OR psyclit OR cinahl OR (psycinfo not "psycinfo database") OR "web of science" OR scopus OR embase)) OR (AB (medline OR pubmed OR psyclit OR cinahl OR (psycinfo not "psycinfo database") OR "web of science" OR scopus OR embase)) OR (TI (meta-analy* OR metaanaly*)) OR (AB (meta-analy* OR metaanaly*)) 21,751

S2 ( DE "ACTIVITIES of daily living" OR DE "QUALITY of life" OR DE "SURVIVAL" OR DE "MORTALITY" ) OR TI ( Activities of Daily Living OR ADL OR Daily Living Activities OR Daily Living Activity OR Chronic Limitation of Activity OR Independent Living OR "Aging in Place" OR Self-Management OR "Quality of Life" OR Life Quality OR Instrumental OR Life Support OR "Prolongation of Life" OR Life Prolongation OR Extraordinary Treatment* OR Longitudinal OR Long-Term OR Longterm OR Survival* OR Mortalit* OR Fatalit* OR Death* OR Cox Model* OR Hazard Model* OR Hazards Model* OR Kaplan-Meier OR Disabilit* OR Work* OR Vocation* OR DALY* OR QALY* OR QUALY* OR Quality Adjusted Life Year* OR Healthy Years Equivalent* OR Adjusted Life Year* OR Satisfaction OR Morbidit* OR Productiv* OR Employment OR Occupational Status OR Unemployment OR Participation OR Engagement OR Function* OR Outcome* OR Care OR "Back to School" OR ((Return* OR Resum*) N1 (School* OR Education OR Study)) OR ((School* OR Education OR Study) N1 (Return* OR Resum*)) ) OR AB ( Activities of Daily Living OR ADL OR Daily Living Activities OR Daily Living Activity OR Chronic Limitation of Activity OR Independent Living OR "Aging in Place" OR Self-Management OR "Quality of Life" OR Life Quality OR Instrumental OR Life Support OR "Prolongation of Life" OR Life Prolongation OR Extraordinary Treatment* OR Longitudinal OR Long-Term OR Longterm OR Survival* OR Mortalit* OR Fatalit* OR Death* OR Cox Model* OR Hazard Model* OR Hazards Model* OR Kaplan-Meier OR Disabilit* OR Work* OR Vocation* OR DALY* OR QALY* OR QUALY* OR Quality Adjusted Life Year* OR Healthy Years Equivalent* OR Adjusted Life Year* OR Satisfaction OR Morbidit* OR Productiv* OR Employment OR Occupational Status OR Unemployment OR Participation OR Engagement OR Function* OR Outcome* OR Care OR "Back to School" OR ((Return* OR Resum*) N1 (School* OR Education OR Study)) OR ((School* OR Education OR Study) N1 (Return* OR Resum*)) ) 456,678

S1 ( DE "BRAIN injuries" OR DE "CEREBRAL hemorrhage" OR DE "HEAD injuries" ) OR TI ( (Brain* N1 (Injur* OR Trauma* OR Posttrauma*)) OR ((Posttrauma* OR Trauma* OR Injur*) N1 Brain*) ) OR AB ( (Brain* N1 (Injur* OR Trauma* OR Posttrauma*)) OR ((Posttrauma* OR Trauma* OR Injur*) N1 Brain*) ) 7,793
