# Supplementary material for: The Australian Traumatic Brain Injury Initiative: Review and Recommendations for Outcome Measures for Use With Adults and Children After Moderate-to-Severe Traumatic Brain Injury
Source: Neurotrauma Rep. 2024 Apr 11;5(1):387–408. doi: 10.1089/neur.2023.0127 (PMC11035854; doi:10.1089/neur.2023.0127)
Supplement: Supplemental data [file Suppl_TableS1.docx]

Supplemental Materials

Table 1. Seventy-five outcome measures extracted as part of the systematic review process.

| Broad domain measured by scale | Measure Name | Abbreviation |
| --- | --- | --- |
| Functional Outcome | The Adaptive Behaviour Assessment System | ABAS |
| ADL | Assessment of Living Skills and Resources | ALSAR |
| Functional Outcome | Barthel Index | BI |
| Participation | Brain Injury Community Rehabiltiation Outcome Scale | BICRO |
| Care | Care and Needs Scale | CANS |
| Participation | Children’s Assessment of Participation and Enjoyment | CAPE |
| Care | Care Hours | CareHours |
| Participation | Child and Adolescent Scale of Participation | CASP |
| Participation | Child Behaviour Checklist | CBCL |
| Participation | Craig Handicap Assessment and Reporting Technique /Short Form/ Revised | CHART(-SF)(R) |
| Global Outcome | Child Health Questionnaire | CHQ |
| Participation | Community Integration Measure | CIM |
| Participation | Community Integration Questionnaire | CIQ |
| Participation | Caregiver Information and Support Link Questionnaire | CISLQ |
| Participation | Canadian Occupational Performance Measure | COPM |
| Participation | Community Outcome Scale | COS |
| Participation | Community Reintegration Scale for Service Members | CRIS |
| Participation | Disability Rating Scale | DRS |
| Functional Outcome | Euorpean Brain Injury Assessment Chart | EBIAC |
| Functional Outcome | European Brain Injury Questionnaire | EBIQ |
| Functional Outcome | Early Functional Abilities | EFA |
| Quality of Life | EuroQol-5 Dimensions, five-level version | EQ-5D-5L |
| Functional Outcome | Functional Independence Measure | FIM |
| Functional Outcome | Functional Status Examination | FSE |
| Functional Outcome | Functional Status II | FSII |
| Functional Outcome | Glasgow Outcome Scale Modified for Chidren | GOS Peds |
| Functional Outcome | Glasgow Outcome Scale/ Glasgow Outcome Scale Extended | GOS/ GOSE |
| Global Outcome | Health Status Questionnaire | HSQ |
| Functional Outcome | Health Utilities Index-Mark | HUI |
| Functional Outcome | Lawton and Brody Instrumental Activities of Daily Living | IADL |
| QoL | Katz Adjustment Scale | KAS |
| QoL | Kinder Lebensqualita ̈tsfragebogen: Children’s Quality of Life Questionnaire-revised | KINDL-R |
| Functional Outcome | King's Outcome Scale for Childhood Head Injury | KOSCHI |
| Functional Outcome | Karnofsky Performance Scale | KPS |
| QoL | Life-3 | Life-3 |
| Participation | Life Habits Questionnaire | LIFE-H |
| QoL | Leisure Satisfaction Scale | LSS |
| Functional Outcome | Mayo Portland Adaptability Inventory/ Portland Adaptability Inventory | MPAI/ PAI |
| Functional Outcome | Modified Rankin Scale | MRS |
| ADL | Nottingham Extended Activities of Daily Living | NEADLI |
| QoL | Neuro-QOL | Neuro-QOL |
| Global Outcome | Nottingham Health Profile | NHP |
| Global Outcome | Neurobehavioural Functioning Inventory | NFI |
| Global Outcome | Neurobehavioural Symptom Inventory | NSI |
| Participation | Participation Assessment with Recombined Tools-Objective | PART-O |
| Functional Outcome | Patient Competency Rating Scale | PCRS |
| Functional Outcome | Paediatric Evaluation of Disability Inventory | PEDI |
| QoL | Pediatric Quality of Life Inventory | PedsQL |
| QoL | Patient Generated Index | PGI |
| Functional Outcome | Paediatric Injury Functional Outcome Scale | PIFOS |
| QoL | Perceived Quality of Life Scale | PQOL |
| QoL | Quality of Life Inventory | QOLI |
| QoL | Quality of Life After Brain Injury | QOLIBRI |
| QoL | Quality of Well-Being | QWB |
| Participation | Role Checklist | RC |
| Participation | Return to Study | RTS |
| Participation | Return to Work | RTW |
| QoL | SARAH quality of life questionnaire for children and adolescents. | SARAH-QoL |
| Functional Outcome | SARAH Physical-Functional Classification of the Child and Adolescent | SARAH-PF |
| Functional Outcome | Social Adjustment Scale | SAS |
| QoL | SmithKline Beecham Quality of Life Scale | SBQoL |
| Global Outcome | Strengths and Difficulties Questionnaire | SDQ |
| QoL | 12-Item Short Form Survey | SF-12 |
| QoL | 36-Item Short Form Survey | SF-36 |
| Functional Outcome | Woodcock Johnson Scales of Independant Behaviour | SIB(-R) |
| QoL | Sickness Impact Profile | SIP |
| Participation | Sydney Psychosocial Reintegration Scale | SPRS |
| Participation | Sydney Psychosocial Reintegration Scale - Children | SPRS-C |
| QoL | Subjective Quality of Life Profile | SQLP |
| Care | Supervision Rating Scale | SRS |
| Participation | Satisfaction With Life Scale | SWLS |
| QoL | Traumatic Brain Injury Quality of Life | TBI-QoL |
| Functional Outcome | Vineland Adaptive Behaviour Scales | VABS |
| Functional Outcome | Functional Independence Measure for Children | WeeFIM |
| Quality of Life | World Health Organisation Quality of Life Scale (Abbreviated) | WHOQOL-BREF |
