# Supplementary material for: The Australian Traumatic Brain Injury Initiative: Review and Recommendations for Outcome Measures for Use With Adults and Children After Moderate-to-Severe Traumatic Brain Injury
Source: Neurotrauma Rep. 2024 Apr 11;5(1):387–408. doi: 10.1089/neur.2023.0127 (PMC11035854; doi:10.1089/neur.2023.0127)
Supplement: Supplemental data [file Suppl_TableS2.docx]

**Table 2.** Additional Specific Measures Considered by Expert Group

| **Participant Group** | **Measure Name (Abbreviation)** |
| --- | --- |
| Adult | Box and Block Test |
|  | Brief Test of Adult Cognition |
|  | Fatigue Severity Scale |
|  | Hospital Anxiety and Depression Scale |
|  | The Latrobe Communication Questionnaire |
|  | Northwick Park Dependency Scale |
|  | Overt Behaviour Scale |
|  | Physical Activity Vital Signs |
| Adult & Paediatric | High Level Mobility Assessment Tool |
|  | 10 metre walk test |
| Paediatric | Child Language Screener |
|  | PedsQL Multidimensional Fatigue Scale |
|  | VINELAND Communication subscale |
|  | VINELAND Motor subscale |
